# Uncoupling Transcription from Covalent Histone Modification

**DOI:** 10.1371/journal.pgen.1004202

**Published:** 2014-04-10

**Authors:** Hesheng Zhang, Lu Gao, Jayamani Anandhakumar, David S. Gross

**Affiliations:** Department of Biochemistry and Molecular Biology, Louisiana State University Health Sciences Center, Shreveport, Louisiana, United States of America; Memorial Sloan-Kettering Cancer Center, United States of America

## Abstract

It is widely accepted that transcriptional regulation of eukaryotic genes is intimately coupled to covalent modifications of the underlying chromatin template, and in certain cases the functional consequences of these modifications have been characterized. Here we present evidence that gene activation in the silent heterochromatin of the yeast *Saccharomyces cerevisiae* can occur in the context of little, if any, covalent histone modification. Using a SIR-regulated heat shock-inducible transgene, *hsp82-2001*, and a natural drug-inducible subtelomeric gene, *YFR057w*, as models we demonstrate that substantial transcriptional induction (>200-fold) can occur in the context of restricted histone loss and negligible levels of H3K4 trimethylation, H3K36 trimethylation and H3K79 dimethylation, modifications commonly linked to transcription initiation and elongation. Heterochromatic gene activation can also occur with minimal H3 and H4 lysine acetylation and without replacement of H2A with the transcription-linked variant H2A.Z. Importantly, absence of histone modification does not stem from reduced transcriptional output, since *hsp82-ΔTATA*, a euchromatic promoter mutant lacking a TATA box and with threefold lower induced transcription than heterochromatic *hsp82-2001*, is strongly hyperacetylated in response to heat shock. Consistent with negligible H3K79 dimethylation, *dot1Δ* cells lacking H3K79 methylase activity show unimpeded occupancy of RNA polymerase II within activated heterochromatic promoter and coding regions. Our results indicate that large increases in transcription can be observed in the virtual absence of histone modifications often thought necessary for gene activation.

## Author Summary

The proper regulation of gene expression is of fundamental importance in the maintenance of normal growth and development. Misregulation of genes can lead to such outcomes as cancer, diabetes and neurodegenerative disease. A key step in gene regulation occurs during the transcription of the chromosomal DNA into messenger RNA by the enzyme, RNA polymerase II. Histones are small, positively charged proteins that package genomic DNA into arrays of bead-like particles termed nucleosomes, the principal components of chromatin. Increasing evidence suggests that nucleosomal histones play an active role in regulating transcription, and that this is derived in part from reversible chemical (“covalent”) modifications that take place on their amino acids. These histone modifications create novel surfaces on nucleosomes that can serve as docking sites for other proteins that control a gene's expression state. In this study we present evidence that contrary to the general case, covalent modifications typically associated with transcription are minimally used by genes embedded in a specialized, condensed chromatin structure termed heterochromatin in the model organism baker's yeast. Our observations are significant, for they suggest that gene transcription can occur in a living cell in the virtual absence of covalent modification of the chromatin template.

## Introduction

Transcription in eukaryotes occurs in the context of chromatin. At most genes, transcriptional activation is accompanied by alterations to the chromatin template, exemplified by the enhanced DNase I sensitivity of coding regions and the presence of associated DNase I hypersensitive sites at linked regulatory elements [1], [2]. DNase I hypersensitivity of promoter regions primarily arises from the partial or transient occupancy of nucleosomes (i.e., nucleosome depletion) [3], [4], while the enhanced nuclease sensitivity of coding regions arises, in large part, from post-translational modification (PTM) of histones. Lysine acetylation of histones increases the accessibility of DNA wrapped around individual nucleosomes [5], contributes to the decondensation of the 30 nm fiber [6], [7] and facilitates nucleosomal displacement during elongation of RNA polymerase [8].

Covalent histone modifications can also create novel nucleosomal surfaces that serve as recognition sites for effector proteins. Histone PTMs, acting singly or in combination, may therefore control the ultimate expression state of a gene, or the ability of the underlying DNA to be repaired, recombined or replicated. This concept has been termed the histone code [9], [10]. A prime example of this is the inducible *INO1* promoter in the budding yeast *S. cerevisiae* where phosphorylation of the Ser10 residue of H3 (H3S10) by the Snf1 kinase triggers acetylation of the Lys14 residue of H3 (H3K14) by the Gcn5 acetyltransferase, and subsequent transcriptional activation [11]. Likewise, in mammalian cells, AMP-activated protein kinase (AMPK)-mediated phosphorylation of H2B S36 has been functionally linked to transcriptional activation of genes responsive to metabolic stress [12]. More recently, the combination of H3K4 trimethylation (H3K4me3) and H4K16 acetylation (H3K16ac) was shown to serve as a high-affinity docking site for the human chromatin remodeling enzyme NURF [13]. Observations such as these have contributed to the widespread idea that histone PTMs play important roles in regulating gene transcription [14], [15].

An alternative view is that histone modifications and the enzymes that impart them are not regulatory; that is, they do not play a causative role in transcription [16]. This arises from the fact that most enzymes that convey histone modifications have no specificity. Moreover, most histone PTMs are short-lived and do not persist in the absence of the proteins that recruit them [16]. Instead, according to this view, histone modifications play other roles. For example, they may assist in the fine-tuning of transcription or in its fidelity. They may also delineate one region of a gene (e.g., promoter) from another (e.g., coding region), as well as from other genetic elements, thereby diversifying the chromatin landscape [17]. Therefore, histone modifications may occur as a consequence, rather than the cause, of dynamic processes such as transcription and nucleosome remodeling [17].

In addition to the precise role(s) played by covalent histone modifications, it is unclear whether the dynamic properties of euchromatin are shared with heterochromatin, the compartment of the nucleus that remains condensed throughout interphase, replicates late, is resistant to recombination, and contains relatively few transcribed genes. In multicellular organisms, heterochromatin can exist in both constitutive and facultative (i.e., regulated) forms. Constitutive heterochromatin is enriched in HP-1 (a structural protein) and SU(VAR)3–9 (an H3K9 methyltransferase) in organisms ranging from fission yeast to mammals, and is characteristic of telomeric and pericentric regions, repetitive DNA elements, and other DNA sequences critical to genomic stability. In contrast, facultative heterochromatin is characteristic of reversibly silenced genes such as X-linked genes in female mammals and genes encoding key developmental regulators, is enriched in H3K27me3- and ubiquitylated H2A-containing nucleosomes, and is under regulation of Polycomb chromatin modification complexes [18], [19], [20].

Budding yeast, although lacking HP-1 and Polycomb proteins, contains specialized chromatin structures that functionally resemble the Polycomb-regulated facultative heterochromatin of insects and vertebrates [21]. These heterochromatic domains are located at telomeres and the *HML* and *HMR* silent mating loci [reviewed in 22], [23]. They are silenced via recruitment of a chromatin modification complex containing the Sir2, Sir3 and Sir4 proteins that horizontally spreads over each telomere or *HM* locus. Absence of any one of the Sir proteins prevents the assembly of silent chromatin [24], [25], [26]. Sir3 and Sir4 are structural proteins [22] while Sir2 is a NAD^+^-dependent lysine deacetylase that deacetylates histones with a preference for H4K16 and H3K56 [27]. Sir2/Sir3/Sir4-mediated silent chromatin resembles the heterochromatin of other organisms in several ways: [i] it is repressive to gene transcription; [ii] it is organized into chromosomal domains that silence in a position-specific rather than sequence-specific fashion; [iii] its assembly involves entry sites that nucleate the formation and spread of repressor proteins; and [iv] the repressed expression state is mitotically inherited from mother to daughter cell [reviewed in 21], [28].

While heterochromatin is generally repressive of transcription, hundreds of genes are localized within this nuclear compartment in protozoa, insects, plants and animals. The *light* gene, and at least eight others in *Drosophila*, depend on a heterochromatic location for normal expression [reviewed in 29]. Likewise, in placental mammals, expression of the *Xist* gene is 100-fold enhanced on the heterochromatic, inactive X chromosome relative to its euchromatic counterpart [reviewed in 30]. In trypanosomes, antigenic variation stems from variegated expression of telomere-silenced surface glycoprotein genes, a phenomenon underlying trypanosomiasis (African sleeping sickness) [reviewed in 31]. Despite the importance of heterochromatic genes, the mechanisms underlying their transcriptional activation remain largely unknown. To gain insight into this, we investigated chromatin alterations that accompany heterochromatic gene activation in *S. cerevisiae*. We found that large increases in the transcription of disparate heterochromatic genes occur in the absence (or near absence) of covalent histone modifications. Strikingly, when the same genes are placed in a euchromatic context, they heavily utilize such modifications.

## Results

### Efficiency of *SIR*-Dependent Silencing Correlates with Targeted Recruitment and Retention of Sir Proteins

**Figure 1:**
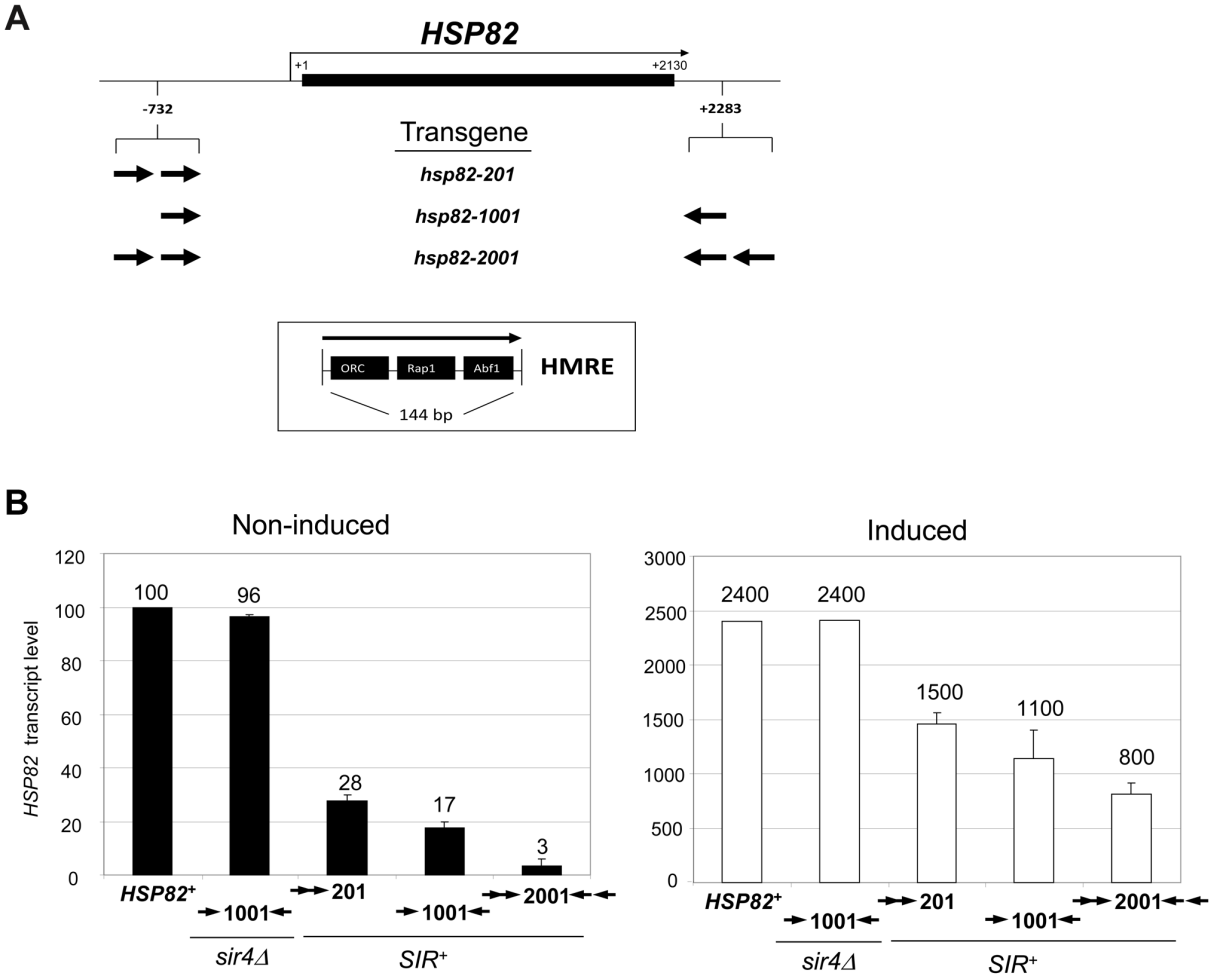
*SIR*-regulated heat shock transgene system. (a) Summary of *hsp82* transgenes used in this study with location, orientation and dosage of integrated *HMRE* silencers indicated by arrows (see Inset for location of silencer binding proteins ORC, Rap1 and Abf1). Transgenes occupy the native chromosomal *HSP82* locus (located ∼95 kb from TEL16L) and contain the indicated silencer insertions with no extraneous DNA sequence (see Materials and Methods). Note that the transcription start site lies 60 bp upstream of the start codon and the 3′ integration site lies ∼50 bp 3′ of the mapped transcription termination site [74]. The ORF is indicated as a black rectangle; coordinates are numbered relative to the ATG codon. (b) Transcriptional output of *hsp82* transgenes under non-inducing (30°C) and inducing conditions (20 min heat shock at 39°C). Depicted are bar graph summaries of Northern analyses of *SIR^+^* and *sir4Δ* cells bearing the indicated *hsp82* transgenes (arrows symbolize integrated silencers as in A); *HSP82* ^+^ was analyzed in the parental strain. Transcript abundance was normalized to *ACT1* and is presented relative to the non-induced *HSP82* ^+^ level set at 100 (depicted are means ± S.D.; N = 2).

To investigate changes in histone abundance and modification state that take place in activated heterochromatin, we took advantage of a previously described heat shock-inducible transgene system [32], [33]. The system consists of the native *HSP82* gene and chromosomal *HSP82* alleles flanked by integrated copies of the *HMRE* mating-type silencer that differ in their dosage and arrangement (illustrated in Figure 1A). As a consequence, the basal transcription of these transgenes is differentially silenced, from 3-fold for *hsp82-201* bearing two upstream silencers to 30-fold for *hsp82-2001* flanked by tandem silencers (see Figure 1B, left). The transgene termed *hsp82-1001*, flanked by single silencers, represents an intermediate case and is ∼6-fold silenced.

**Figure 2:**
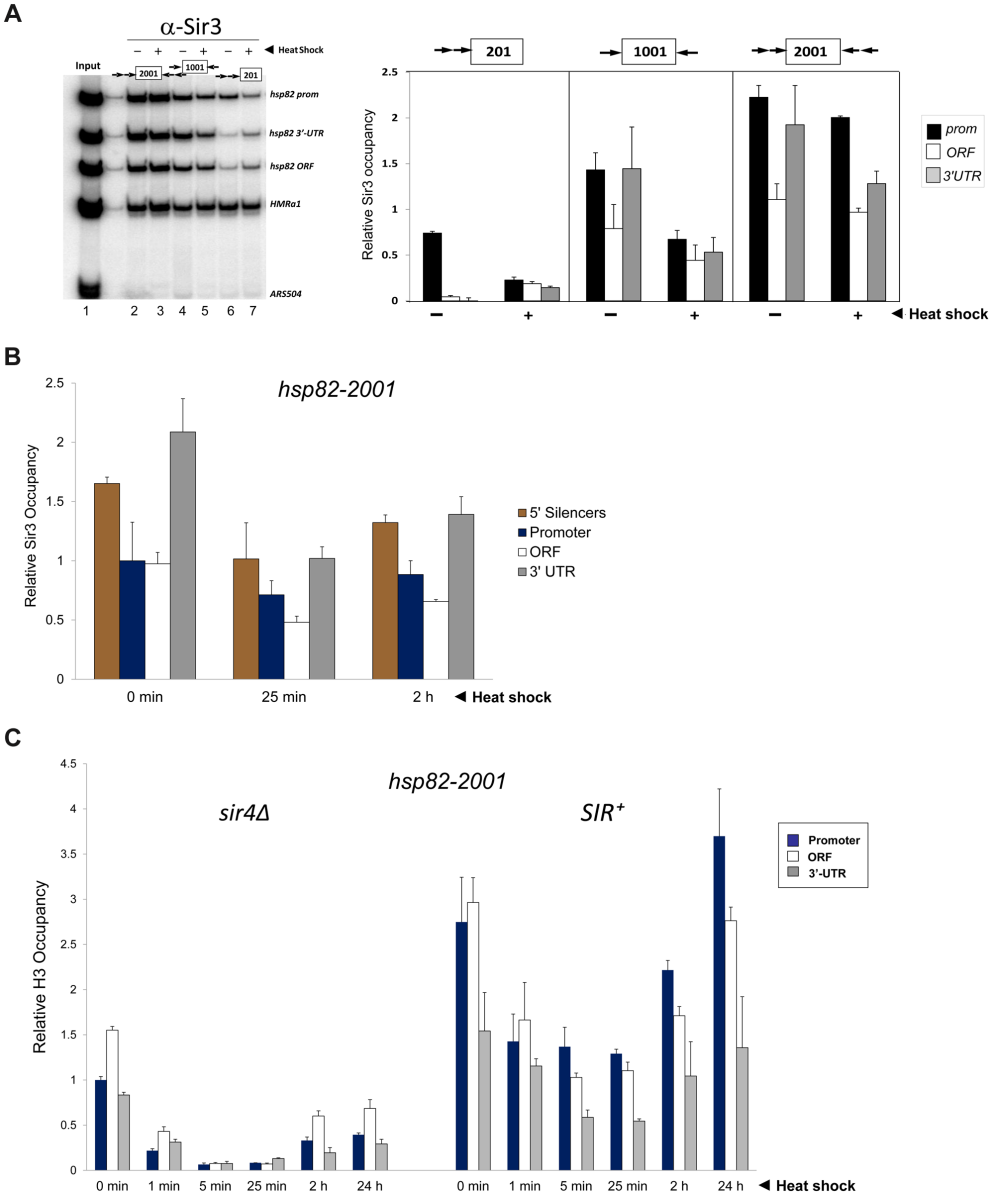
Retention of the Sir protein complex and increased nucleosome density and stability at the heat shock-induced *hsp82-2001* transgene. (a) *In vivo* crosslinking analysis of Sir3 at the promoter, ORF and 3′-UTR of *hsp82-201*, *hsp82-1001* and *hsp82-2001*. Crosslinked chromatin, sheared to a mean size of ∼0.5 kb, was isolated from cells cultivated at 30°C and either maintained at that temperature or subjected to a 20 min 39°C heat shock (HS) (− and +, respectively). Following immunoprecipitation, crosslinks were reversed and purified DNA was subjected to quantitative multiplex PCR in the presence of [α-^32^P] dATP using primers specific for the five loci indicated. A gel analysis of multiplex PCR products is presented on the left, while a summary of three independent experiments (means ± S.E.) is presented on the right. Input sample (lane 1), derived from strain EAS2011, represents 4% of soluble chromatin used in the corresponding IP (lane 2). In the histogram, Sir3 occupancy at each *hsp82* transgene was normalized to its occupancy at *HMRa1*. prom, promoter. (b) ChIP analysis of Sir3 at *hsp82-2001* as in A, except that cells were subjected to the indicated heat shock time course and quantification of Sir3 abundance was performed using Real Time qPCR. Sir3 abundance at the indicated regions was normalized to its occupancy at *HMRa2*; illustrated are means ± S.D. (N = 2; qPCR = 4). (c) Histone H3 abundance at the promoter, ORF and 3′-UTR of *hsp82-2001* in *sir4Δ* and *SIR^+^* contexts as indicated, normalized to its occupancy at *ARS504*. Cultures were maintained at 30°C (0 min) or subjected to an instantaneous 39°C upshift for the times indicated. Quantification performed as in B; depicted are means ± S.D.; N = 2, qPCR = 4.

As expected, Sir proteins occupy the promoter region of each transgene under non-heat-shock (NHS) conditions, and at levels that roughly correlate with the extent of silencing (see Figure 2, panels A and B for a Sir3 ChIP analysis; similar results were previously seen for Sir2 [24]). Sir3 was also observed within the coding regions of *hsp82-1001* and *hsp82-2001* but not within the ORF of the weakly silenced *hsp82-201* gene. Indeed, the domain of silent chromatin at *hsp82-1001* and *hsp82-2001* spans at least 4 kb based on both ChIP and mRNA expression criteria (Figure S1), closely resembling that seen at the native *HMR* locus [26], [34]. This observation is consistent with the idea that spread of the Sir2/3/4 complex from its site of recruitment is antagonized by the presence of enhancer and promoter sequences which serve as boundary elements [35], [36].

In response to a 20 min heat shock (HS), all three transgenes were strongly activated (Figure 1B, right). Notably, despite >200-fold activation, Sir3 occupancy within the *hsp82-2001* locus was only slightly reduced (Figure 2, panels A and B), contrasting with the less efficiently silenced transgenes where Sir3 localization was altered through either dispersal or dissociation (Figures 2A and S1A, compare HS (+) samples with NHS (−)). Thus, transcriptional activation of the most efficiently silenced transgene occurred with minimal loss of the Sir2/3/4 complex at either promoter or coding region, consistent with earlier observations of the *hsp82-2001* promoter and recent observations of an activated subtelomeric *URA3* gene [24], [37].

Disruption of silent chromatin through *SIR4* deletion restored transcript levels of each transgene to WT levels (Figure 1B, *hsp82-1001 sir4Δ* strain and data not shown). This indicates that cis-acting *HMRE* silencers (and the sequence-specific proteins bound to them) do not affect *HSP82* regulation; assembly of the Sir protein complex is required. Consistent with this, the promoter chromatin structure of the three transgenes in a *sir4Δ* background is indistinguishable from the euchromatic *HSP82* gene, based on nucleotide-resolution DNase I, MNase and dimethyl sulfate genomic footprint analyses [33].

### Nucleosomal Density Is Increased across the Hyperrepressed *hsp82-2001* Transgene and Heat Shock-Induced Histone Eviction Is Suppressed

We next investigated the effect of the Sir proteins on nucleosome density and stability. As previously seen for wild-type *HSP82* [38], histone H3 was rapidly depleted in response to heat shock of the euchromatic *hsp82-2001* gene, exhibiting a 75% reduction within 60 sec and >90% reduction within 5 min (Figure 2C, *sir4Δ* samples). Consistent with diminished transcriptional activity during chronic heat shock [39], [40], H3 levels were partially restored between 2 and 24 hr. Assembly of *HSP82* into heterochromatin not only resulted in at least a twofold increase in nucleosomal density throughout the gene (compare *SIR^+^* with *sir4Δ* cells, 0 min), but also restricted the dynamic nature of the chromatin as the extent of nucleosomal disassembly was substantially reduced. Virtually identical results were seen when abundance of either myc-H4 or H2A was evaluated (data not shown); therefore, large (>200-fold) increases in expression can take place in the context of relatively modest changes in nucleosome occupancy, consistent with the retention of the Sir2/3/4 complex described above.

### The Transcriptional Machinery Dynamically Associates with Heterochromatic *hsp82-2001*

**Figure 3:**
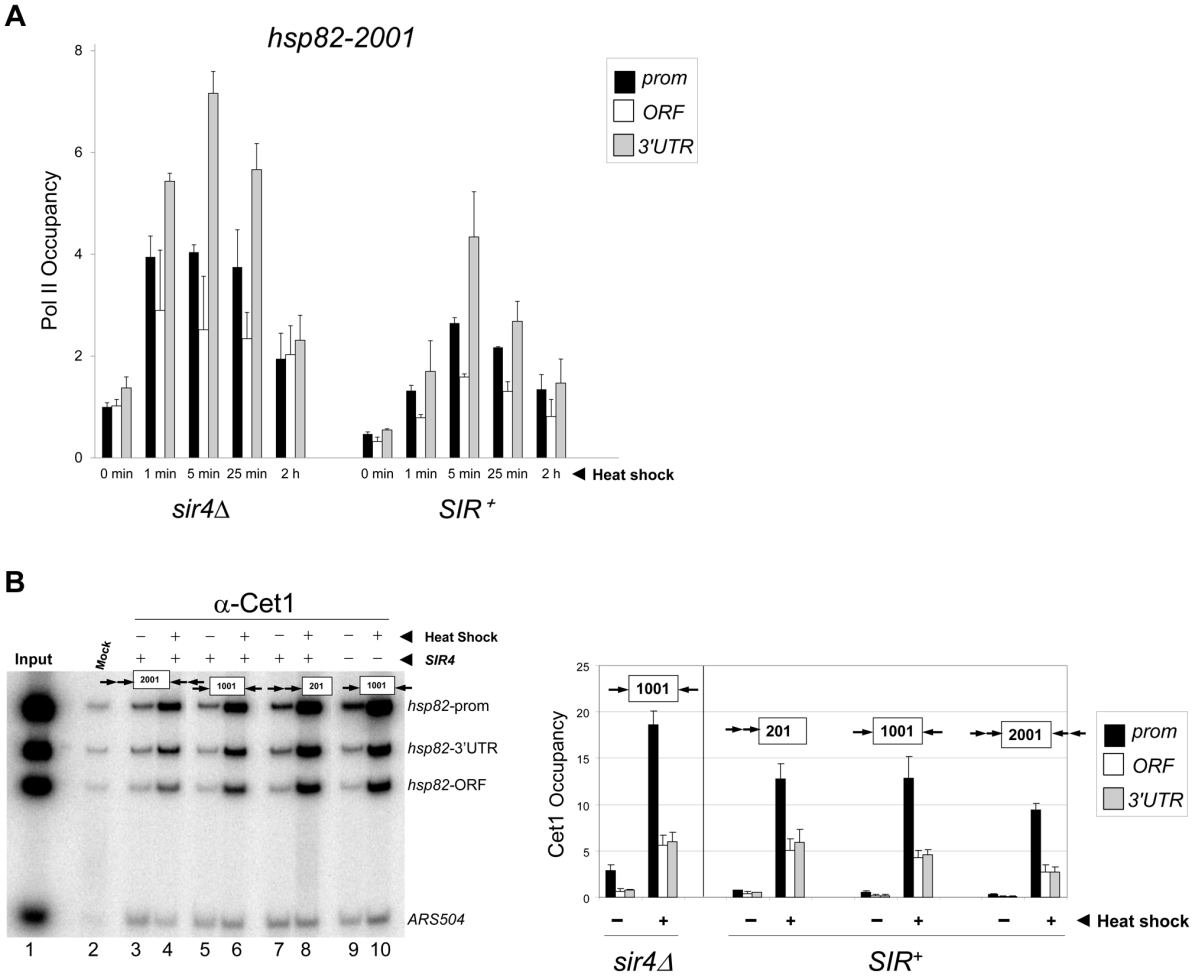
Components of the transcriptional machinery are dynamically recruited to activated heterochromatic genes. (a) ChIP-qPCR analysis of Pol II occupancy at the promoter, ORF and 3′-UTR of the *hsp82-2001* gene in *sir4Δ* and *SIR^+^* cells heat shocked for the indicated times. All values represent net ChIP signals (immune – pre-immune serum) at *hsp82-2001* normalized to those at *ARS504*. Pol II occupancy at the euchromatic *hsp82-2001* promoter was set to 1.0; all other occupancies are scaled relative to this. (b) ChIP analysis of Cet1 occupancy of *hsp82-201*, *hsp82-1001* and *hsp82-2001* under NHS (−) and 20 min HS (+) conditions, conducted as in Figure 2A. Left, gel analysis. Input sample (lane 1) represents 0.4% of soluble chromatin used in each IP. Mock, chromatin reacted with beads alone. Lanes 3–10, chromatin isolated from *SIR^+^* and *sir4Δ* strains as indicated (+ and −, respectively) and immunoprecipitated with a Cet1-specific antibody. Histogram represents net mean values (immune signal minus beads alone) of three independent experiments.

Given the relatively static state of the chromatin, we next asked whether inducible occupancy of the Pol II transcriptional machinery could be detected at the heterochromatic *hsp82-2001* transgene. Consistent with earlier observations of *SIR*-silenced genes [24], [41], [42], [43] and reconstituted SIR-heterochromatin [44], Pol II was detectable within the promoter region of non-induced *hsp82-2001*, albeit at a reduced level (Figure 3A, *SIR^+^*, 0 min). Importantly, its occupancy was substantially enhanced (10- to 15-fold) by heat shock. We additionally examined occupancy of the capping enzyme Cet1. Previous analysis of the *HMLα1/α2* and *HMRa1* silent mating genes indicated that occupancy of Cet1 was strongly restricted under *SIR*-silencing conditions [42], consistent with the notion that *SIR* elicits silencing, at least in part, by targeting steps downstream of PIC assembly. In support, we found that under non-inducing conditions Cet1 was at near-background levels at all three heterochromatic *hsp82* transgenes (Figure 3B). This restriction was dramatically overridden by heat shock, which resulted in a >30-fold increase in Cet1 occupancy of the 5′-end of *hsp82-2001* and similar increases within the 5′-ends of *hsp82-201* and *hsp82-1001*. Therefore, at least two components of the basic transcriptional machinery, Pol II and Cet1, dynamically occupy the hyperrepressed *hsp82-2001* transgene in response to heat shock, in contrast to either histones or Sir proteins.

### *SIR* Severely Reduces H3 and H4 N-Terminal Acetylation upon Transcriptional Activation

**Figure 4:**
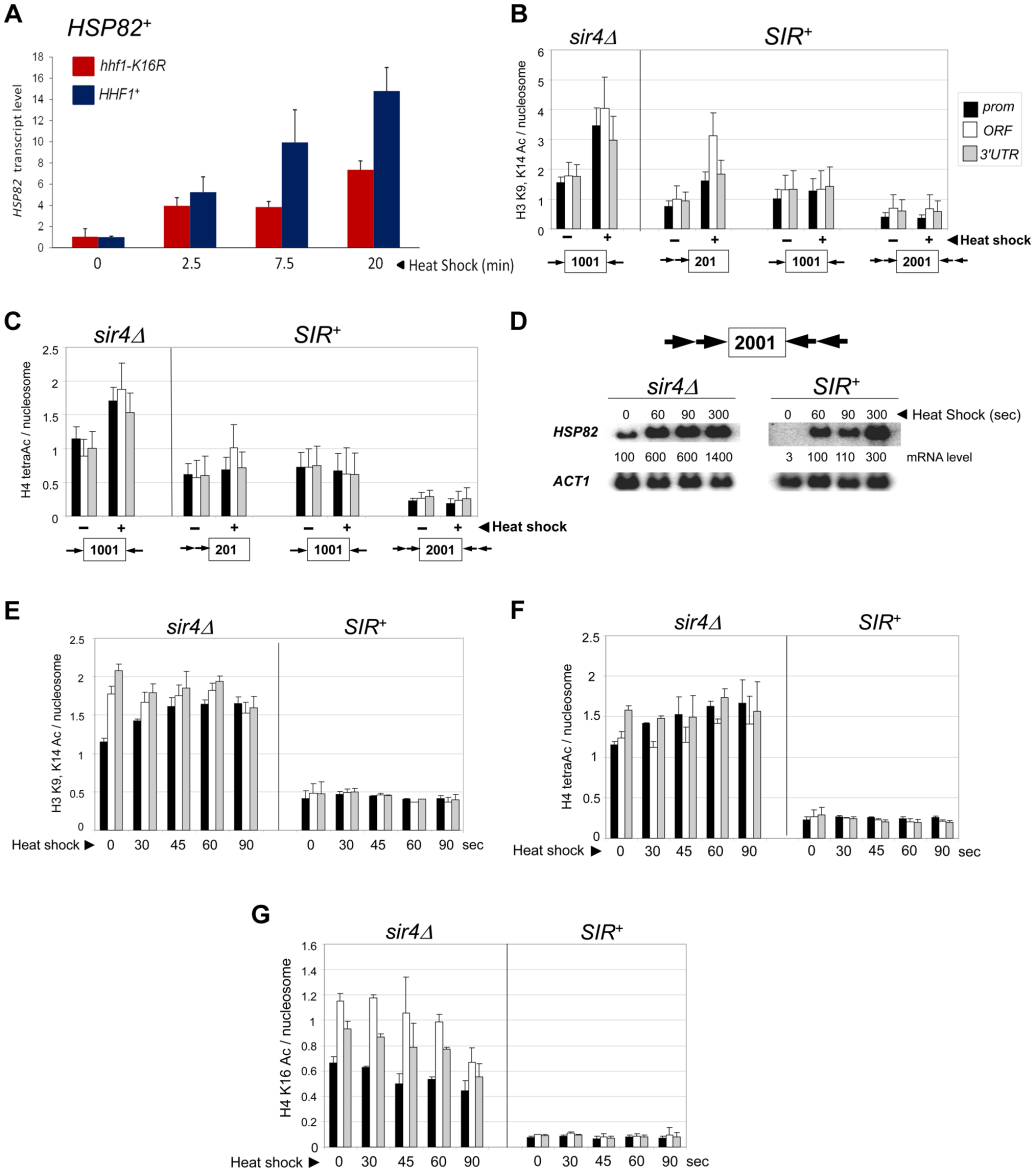
Heterochromatic gene activation occurs in the context of negligible H3 and H4 acetylation. (a) Induced transcription of the wild-type *HSP82* gene is impaired by an H4 K16R mutation. Transcript abundance was assayed in isogenic *HHF1^+^* and *hhf1-K16R* cells subjected to a 30° to 39°C temperature shift for the indicated times. Total RNA was isolated, cDNA synthesized, and *HSP82* levels were quantified by RT-qPCR followed by normalization to *SCR1*. Quotients were scaled to the non-induced *HHF1^+^* sample which was set to 1. Depicted are means ± S.D. (N = 2; qPCR = 4). (b) ChIP analysis of H3 diacetylated at K9, K14 at the indicated *hsp82* transgenes in *sir4Δ* or *SIR^+^* cells cultivated under NHS (−) or 20 min HS (+) conditions using multiplex PCR as in Figure 2A. Shown are quotients of diacetylated H3 signal divided by Myc-H4 signal (means ± S.E.; N = 3). Occupancies of acetylated H3 and Myc-H4 at the *hsp82* transgenes were normalized to their respective occupancies at *PHO5*. (c) As in B, except that a ChIP analysis of tetra-acetylated H4 is depicted. (d) Northern analysis of *hsp82-2001* in *sir4Δ* or *SIR^+^* cells subjected to an instantaneous 30° to 39°C thermal upshift for the times indicated. *HSP82* mRNA levels, normalized to those of *ACT1*, were quantified for each time point (values represent means of two independent experiments). (e) H3 K9ac,K14ac ChIP analysis of *hsp82-2001* in *sir4Δ* or *SIR^+^* cells for the indicated times following instantaneous heat shock. Depicted are diacetylated H3/Myc-H4 quotients quantified as in B. (f) Tetra-acetylated H4 ChIP analysis of *hsp82-2001* as in E. (g) H4K16ac ChIP analysis of *hsp82-2001* as in E.

Previous work has shown that euchromatic *hsp82* alleles are dependent on the histone acetyltransferases Gcn5 and Esa1 for normal transcriptional activation [45]. Consistent with this, we found that *HSP82^+^* activation was impaired in an H4 K16R mutant (Figure 4A). We therefore asked whether acetylation of histones H3 and H4, a hallmark of gene activation [14], [46], [47], accompanies induction of the *hsp82* transgenes. As shown in Figures 4B and 4C, nucleosomes occupying the euchromatic *hsp82* transgene (*sir4Δ* cells) were both H3 di-acetylated and H4 tetra-acetylated under NHS (−) conditions, mimicking the wild-type *HSP82* gene [38]. By contrast, nucleosomes within the *SIR*-silenced *hsp82-2001* gene were negligibly acetylated under the same conditions. Thus, *hsp82-2001* conforms to the general notion of a histone code, in which absence of N-tail histone acetylation codes for inactivation. The less efficiently silenced transgenes were also impoverished in both di-acetylated H3 and tetra-acetylated H4, but to a lesser degree. Notably, upon heat shock (+), the euchromatic transgene was enriched in both acetylated isoforms (once again resembling *HSP82^+^*) yet there was no detectable enrichment of either at the hyperrepressed *hsp82-2001* gene. Similarly, the moderately silenced *hsp82-1001* transgene, despite >60-fold increase in expression in response to heat shock, showed no enrichment in acetylation. By contrast, the weakly silenced *hsp82-201* gene was H3 hyperacetylated, resembling its euchromatic counterpart.

The foregoing analysis indicates that the minimal acetylation state of heterochromatic *hsp82-2001* remains unaltered following a 20 min heat shock. However, as transcript accumulation is evident within the first 60 sec (see Figure 4D, *SIR^+^* samples), it was possible that transient histone acetylation may have occurred. To address this, we assayed H3 and H4 acetylation of *hsp82-2001* 30 sec following an instantaneous heat shock, and at 15- to 30-sec intervals thereafter, to obtain ‘snapshots’ of the H3/H4 acetylation state within this gene. Despite a 30-fold increase in *hsp82* transcript level within the first 60–90 sec of heat shock, neither di-acetylated H3 nor tetra-acetylated H4 was detectably increased (Figures 4E and 4F; *SIR^+^* background). This again contrasts with the euchromatic state where promoter-associated nucleosomes, already enriched in acetylated H3 and H4 molecules, showed a further ∼30% increase.

**Figure 5:**
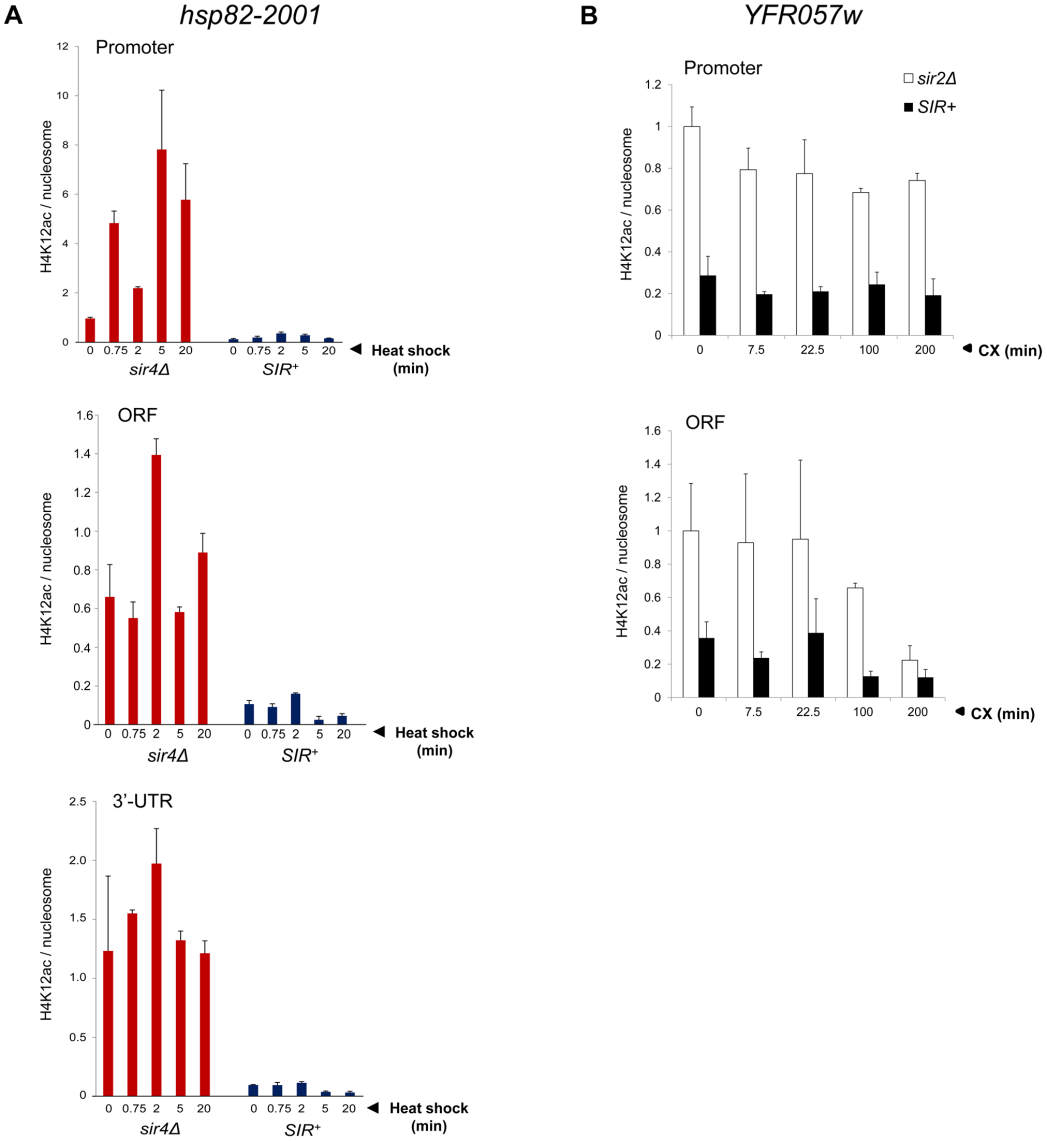
Activation of heterochromatic genes occurs in the context of negligible H4K12 acetylation. (a) H4K12ac enrichment at *hsp82-2001* subjected to heat shock for the indicated times. H4K12Ac and H3 ChIP signals were quantified at promoter, ORF and 3′-UTR using Real Time qPCR and normalized to those measured at *PMA1* and *ARS504* (for H4K12ac and H3, respectively). H4K12ac/H3 quotients were then determined and scaled to the non-induced promoter signal in *sir4Δ* cells, which was set to 1. Depicted are means ± S.D (N = 2; qPCR = 4). (b) H4K12ac enrichment at *YFR057W* promoter and ORF at the indicated times following addition of 200 µg/ml cycloheximide. Quantification was performed as in A.

We separately examined H4K16 acetylation, given that this modification is sufficient to inhibit formation of the compact 30 nm fiber *in vitro* [7], and as previously mentioned, is a preferred Sir2 target. As above, there was no increase in H4K16 acetylation, even transiently, as this modification remained at background levels throughout the heat shock (Figure 4G, *SIR^+^*). This finding suggests that Sir2 may actively deacetylate H4K16 to suppress the extent of chromatin unfolding and, as a consequence, diminish gross transcriptional output. We also examined H4K12 acetylation, which has been implicated in telomeric heterochromatin formation and function in *S. cerevisiae* [48]. However, in contrast to euchromatic *hsp82-2001*, the hyperrepressed *hsp82-2001* transgene was assembled in nucleosomes containing only background levels of H4K12ac, with little if any enrichment upon heat shock (Figure 5A). Thus, its role may be telomere-specific (see below).

### Activating H3K56ac and H3K36me3 Marks Are Strongly Suppressed during Heterochromatic Gene Induction

**Figure 6:**
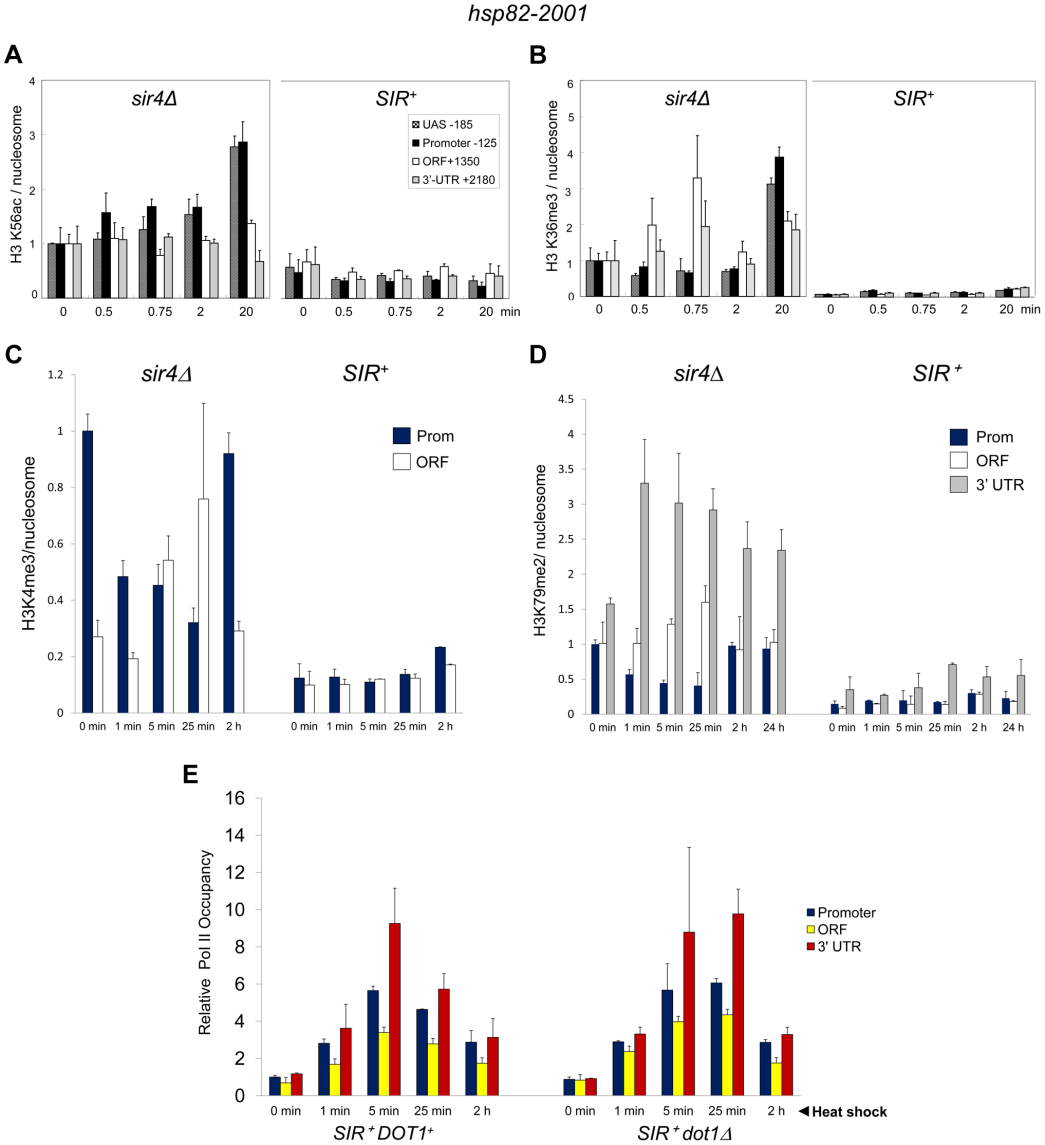
Heterochromatic gene activation occurs in the context of minimal transcription-linked H3 methylation and is unimpaired by ablation of Dot1. (a) H3K56ac ChIP analysis of *hsp82-2001* in *sir4Δ* or *SIR^+^* cells subjected to an instantaneous 30° to 39°C thermal upshift for the times indicated. Quantification was done using Real Time qPCR. The acetylated H3K56/Myc-H4 quotient of the non-induced *sir4Δ* sample was set to 1.0 for each amplicon. PTM-specific and Myc-H4 signals at the heat shock transgene were normalized to those measured at *PMA1* and *ARS504*, respectively. Shown are means ± S.D. (N = 2; qPCR = 4). (b) H3K36me3 ChIP analysis of *hsp82-2001* conducted as in A. (c) H3K4me3 ChIP analysis of *hsp82-2001* in *sir4Δ* or *SIR^+^* cells subjected to an instantaneous 30° to 39°C thermal upshift for the times indicated. Quantification and scaling were done as in A, except H3K4me3/H3 quotients are depicted, and both PTM-specific and H3 signals were normalized to those measured at *ARS504*. Shown are means ± S.D. (N = 2; qPCR = 4). (d) H3K79me2 ChIP analysis of *hsp82-2001* in *sir4Δ* or *SIR^+^* cells as in C. (e) Pol II ChIP analysis of heterochromatic *hsp82-2001* in *DOT1^+^* and *dot1Δ* strains subjected to heat shock as above. Pol II occupancy was determined using ChIP-qPCR as in Figure 3A. Shown are means ± S.D. (N = 2; qPCR = 4).

Since the above results argue that heterochromatic transcription is uncoupled from histone N-terminal lysine acetylation, we asked whether acetylation of K56, located within the globular core of H3, or methylation of K36, located at the junction between the H3 N-terminus and the globular domain, accompanies transcriptional activation. H3K56 acetylation is predicted to break a histone-DNA interaction, potentially destabilizing the nucleosome [49] and is a mark of Pol II elongation due to histone exchange [50]. Indeed, there exists a tight linkage between H3K56 acetylation and nucleosomal disassembly *in vivo* [51]. H3K56 acetylation is of additional interest, given that it is a target of Sir2, and its deacetylation is important in the compaction of telomeric silent chromatin and concomitant repression of transcription [27]. *SIR* reduced H3K56ac levels at non-induced *hsp82-2001* ∼2-fold (Figure 6A). More strikingly, H3K56ac enrichment remained low following a 20 min heat shock time course, when transcript accumulation increased >200-fold. This static modification state contrasts with the euchromatic *hsp82* gene where the already elevated H3K56ac levels exhibited a further 2.5 to 3-fold increase at both UAS and promoter regions (Figure 6A, *sir4Δ*).

H3K36me3 is a hallmark of Pol II elongation within gene coding regions [52]. Despite this, robust transcriptional activation of *hsp82-2001* takes place in the context of minimal H3K36 trimethylation (<5% of that seen in euchromatin; Figure 6B). Thus, similar to H3K56 acetylation, H3K36 methylation is largely suppressed during heterochromatic gene activation.

### H3K4 and H3K79 Methyl Marks Are Likewise Efficiently Suppressed by *SIR*

We next addressed the role of H3 methylation at lysines 4 and 79. H3K4 trimethylation is a broadly conserved covalent modification of active (or potentially active) gene promoters [52] where it can promote transcription by serving as a recognition site for a number of transcriptional co-regulators such as TFIID, SAGA and NuA3 [53]. Di-methylation of H3K79, located within the globular core of H3, is also linked with activation [54] and its presence correlates with enhanced access of transcription factors to chromatin [55]. Consistent with roles in expression, the euchromatic *hsp82* gene is heavily modified with both methyl marks – H3K4me3 particularly within the promoter region and H3K79me2 particularly within the 3′-transcribed region (Figure 6, panels C and D). Strikingly, *SIR* strongly suppressed both H3K4me3 and H3K79me2 enrichment within the *hsp82-2001* transgene throughout a heat shock time course. Moreover, deletion of *DOT1*, which encodes the sole H3K79 methyltransferase in *S. cerevisiae*, had virtually no effect on either Pol II recruitment kinetics or occupancy levels throughout the *hsp82-2001* gene (Figure 6E). This indicates that H3K79 methylation is unnecessary for robust activation of heterochromatic *hsp82-2001*, in contrast to observations of a telomeric *URA3* gene which suggested that H3K79me1 and H3K79me2 are important in disrupting transcriptional silencing [37] (see Discussion). These results, together with those described above, argue that a substantial increase in transcription can take place in the context of heightened nucleosomal density and minimal covalent histone modification.

### The Histone Variant H2A.Z Is Dispensable for Activation within Silent Chromatin

**Figure 7:**
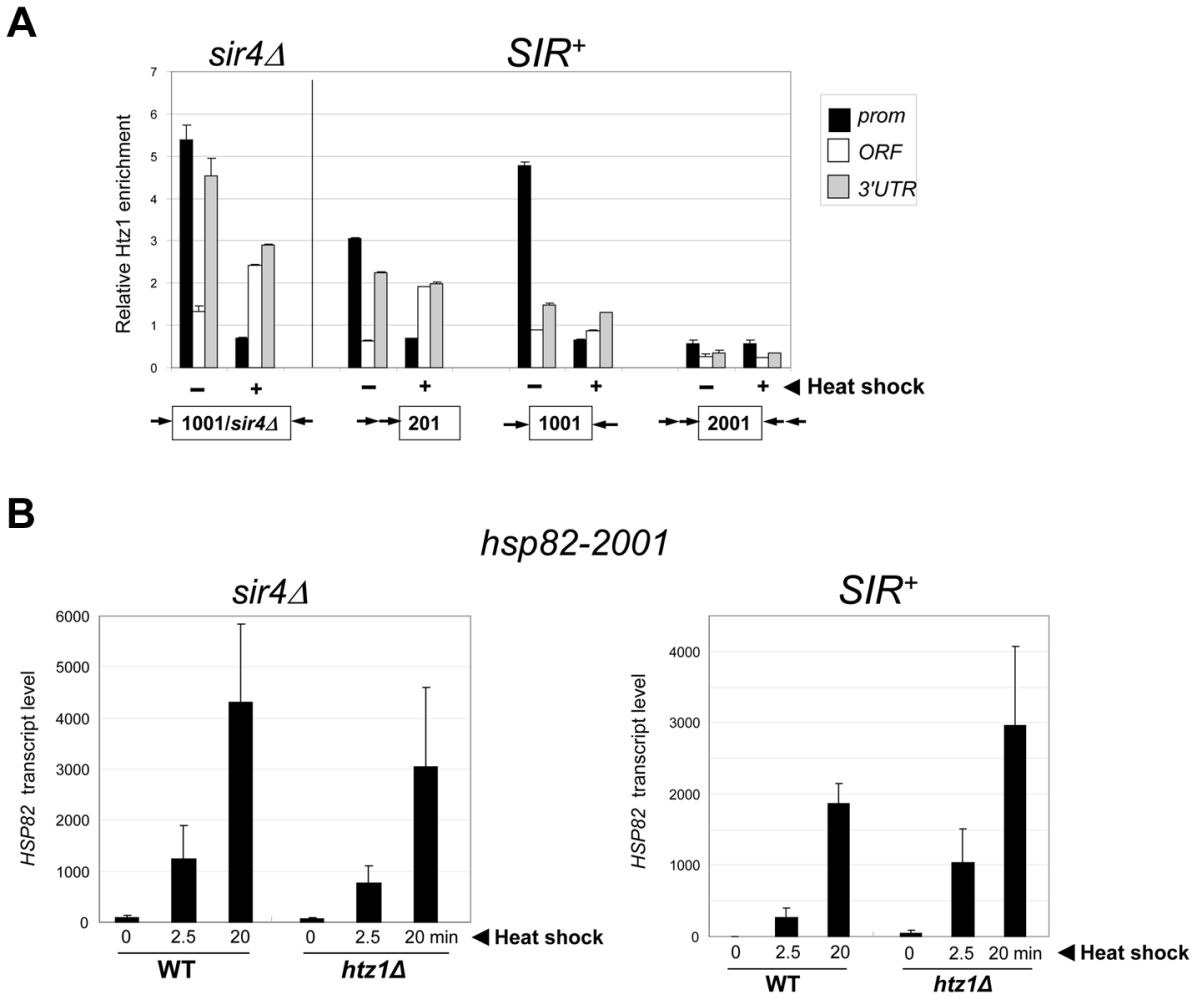
Activation of heterochromatic *hsp82-2001* occurs in the presence of minimal H2A.Z levels and is unimpaired by deletion of *HTZ1*. (a) ChIP analysis of H2A.Z at the indicated *hsp82* alleles under NHS (−) and 20 min HS (+) conditions conducted as in Figure 4B, with H2A.Z content normalized per nucleosome (Myc-H4 abundance). Shown are means ± S.D. (N = 2). (b) *hsp82-2001* transcript abundance was determined in WT and *htz1Δ* cells (*sir4Δ* and *SIR^+^* as indicated) subjected to heat shock for the indicated periods of time. RNA was isolated, cDNA synthesized and *HSP82* transcripts were quantified using RT-qPCR and normalized to *SCR1* as in Figure 4A. Depicted are means ± S.D. (N = 2; qPCR = 4).

We next examined the role of the histone variant H2A.Z (Htz1 in *S. cerevisiae*). Replacement of canonical H2A with H2A.Z at the +1 and or −1 nucleosome poises promoters for transcriptional activation as H2A.Z-containing nucleosomes are more susceptible to disassembly than canonical nucleosomes [56], [57]. ChIP analysis revealed that H2A.Z is enriched within the euchromatic *hsp82* promoter, consistent with prior findings [56], and is preferentially evicted upon heat shock (Figure 7A, *hsp82-1001 sir4Δ*, black bars). As expected, H2A.Z abundance was reduced at the *SIR*-repressed transgenes. At *hsp82-201*, its abundance was reduced 40% at the promoter, while at *hsp82-1001*, H2A.Z levels were altered only at the 3′-end. At both genes, promoter-associated H2A.Z was drastically reduced concomitant with transcriptional induction (Figure 7A, (+) samples). Therefore, the promoter regions of these partially silenced transgenes, like their euchromatic counterparts, are assembled into H2A.Z-containing nucleosomes that are preferentially displaced in response to heat shock. In contrast, the hyperrepressed *hsp82-2001* gene is nearly bereft of H2A.Z in non-induced cells (∼90% reduced within both promoter and coding region) and this reduced level remained unchanged during activation. Consistent with this observation, deletion of the gene encoding H2A.Z did not impair *hsp82-2001* mRNA induction, and may have even enhanced it (Figure 7B, *SIR^+^ htz1Δ* samples).

### Euchromatic *hsp82-ΔTATA* Undergoes Substantial Chromatin Modification in Response to Heat Shock

**Figure 8:**
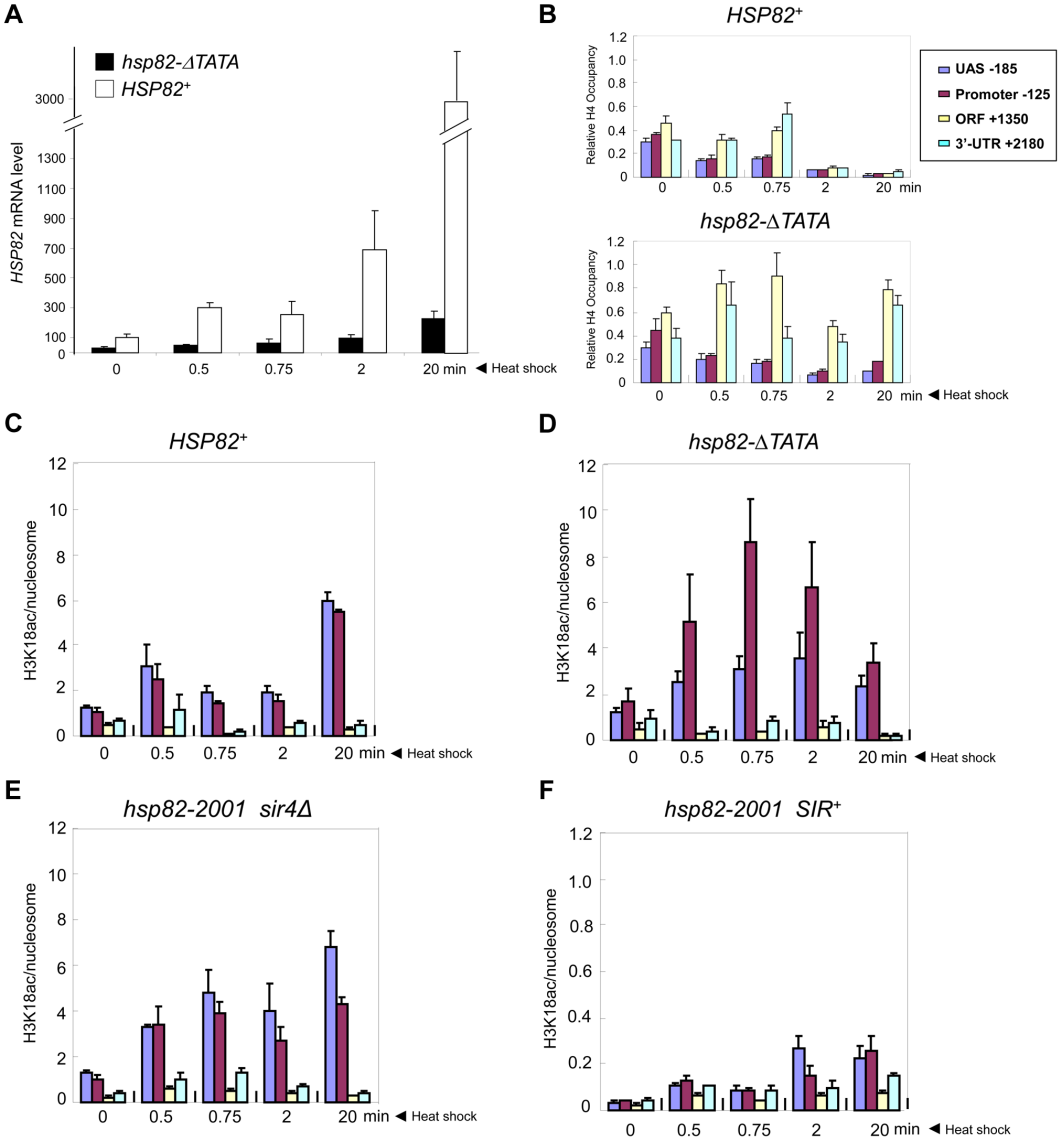
Euchromatic *hsp82-ΔTATA* undergoes nucleosomal disassembly and H3 hyperacetylation within its 5′-regulatory region. (a) Expression analysis of isogenic strains bearing either *HSP82* or *hsp82-ΔTATA* (SLY101 background) and subjected to a heat shock time course for the indicated times. *hsp82* transcript levels were quantified and normalized as in Figure 4A (N = 2; qPCR = 4). (b) Summary of Myc-H4 occupancy at the indicated regions within *HSP82* and *hsp82-ΔTATA*, normalized to its occupancy at *ARS504*. Quantification was done as in Figure 2C (N = 2; qPCR = 4). (c) H3K18 acetylation analysis of *HSP82* over a 20 min heat shock time course as in Figure 5A (means ± S.D.; N = 2; qPCR = 4). (d) H3K18ac analysis of *hsp82-ΔTATA*, performed as in C. (e) H3K18ac analysis of euchromatic *hsp82-2001* (*sir4Δ* cells). (f) H3K18ac analysis of heterochromatic *hsp82-2001* (*SIR^+^* cells) (note difference in scale).

It could be argued that the near-absence of covalent histone modification at *hsp82-2001* is a consequence of its lower transcriptional output relative to wild-type *HSP82*. To address this possibility, we used an attenuated euchromatic *hsp82* allele, *hsp82-ΔTATA*, that bears a 19 bp chromosomal substitution of the TATA box and surrounding region [38]. This mutation resulted in a 10-fold reduction in 20 min heat shock-induced transcript levels (Figure 8A). By comparison, silent chromatin diminished the activated expression of *hsp82-2001* only 2- to 3-fold (e.g., see Figures 1B, 7B). If absence of histone PTMs and other chromatin alterations stem from diminished gross transcriptional output, then it would be predicted that *hsp82-ΔTATA* chromatin would not be modified upon its activation. However, heat shock-activated *hsp82-ΔTATA* undergoes H4 displacement and substantial H3 acetylation. H4 depletion within the UAS/promoter of *hsp82-ΔTATA* was both rapid and extensive, closely resembling wild-type *HSP82*, although there was little histone loss over the coding region (Figure 8B), consistent with diminished expression. Moreover, acetylation of H3K18, which is catalyzed by SAGA [58] and strongly correlates with transcriptional activation in euchromatin [59], was particularly prominent within the UAS/promoter region of *hsp82-ΔTATA* (Figure 8D). By contrast, the upstream region of heterochromatic *hsp82-2001* was negligibly modified at H3K18 and with delayed kinetics (Figure 8F; note difference in scale), whereas H3K18 acetylation at euchromatic *hsp82-2001* resembled *HSP82^+^* (compare Figures 8C and 8E). These results argue that the Hsf1-activated heterochromatic *hsp82-2001* gene is transcribed at a sufficiently high level to undergo nucleosomal disassembly and significant H3 acetylation over its promoter-proximal region, yet does not do so.

### Transcriptional Activation of a Natural Subtelomeric Gene Likewise Occurs with Minimal Histone Modification

The foregoing results indicate that transcriptional activation of heterochromatic *hsp82* can occur largely in the absence of nucleosomal modifications that are characteristic of euchromatic genes. While a *SIR*-dependent chromatin structure is present at *hsp82-2001* that, by several criteria, resembles the silent chromatin established at the natively silenced *HM* loci (Figures 2 and S1A) [24], [33], it is possible that the absence (or near-absence) of activating histone modifications is unique to Hsf1-regulated genes. Indeed, retention of epigenetic information at yeast heat shock genes may not be critical given the extent of histone loss that occurs within both regulatory and coding regions upon their activation (Figure 2C) [38], [60].

**Figure 9:**
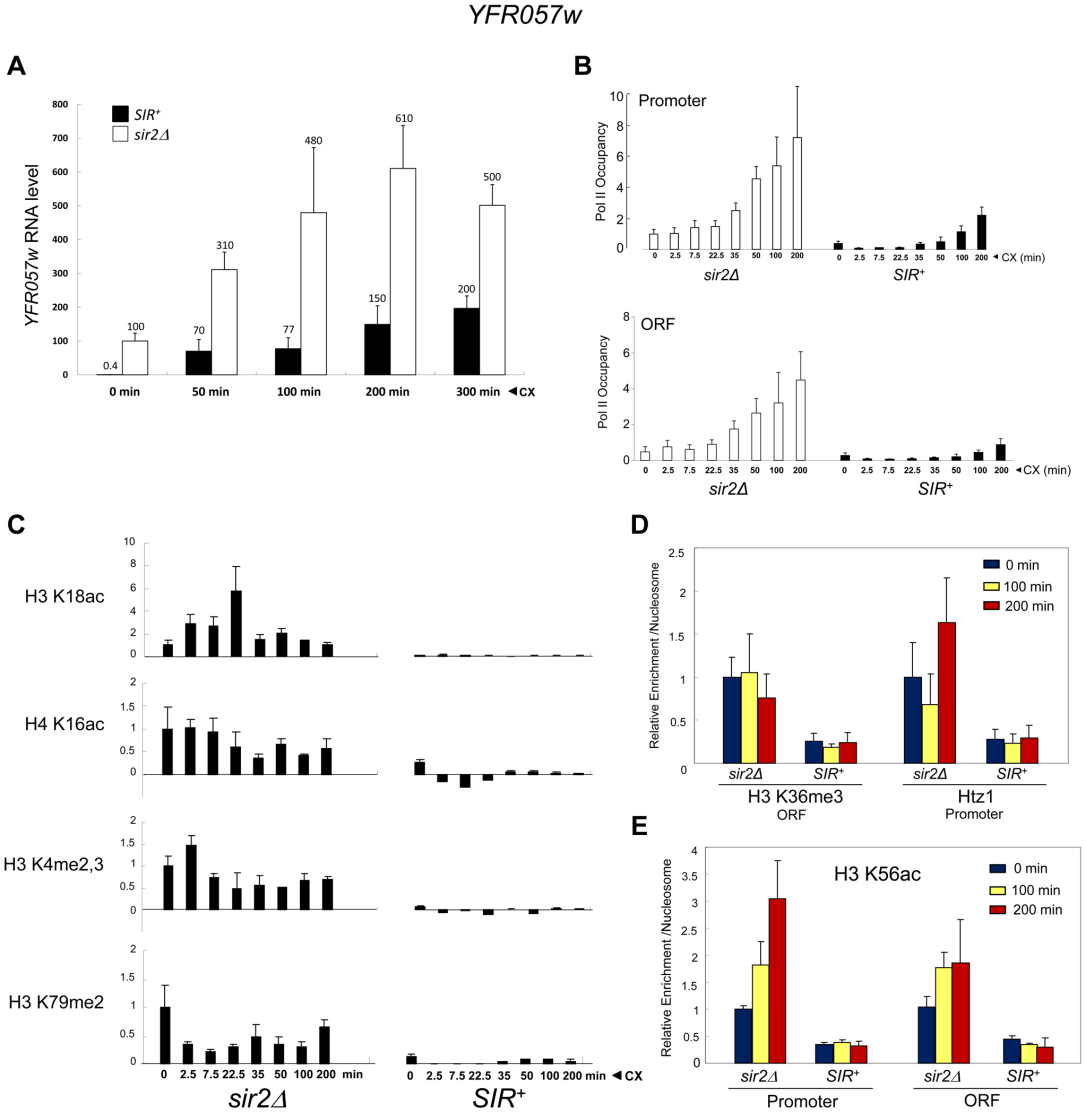
Transcriptional activation of the subtelomeric *YFR057w* gene is unlinked to covalent histone modification. (a) *YFR057w* mRNA levels in *SIR^+^* and *sir2Δ* cells (BY4741 background) exposed to 200 µg/ml cycloheximide (CX) for the indicated times. *YFR057w* transcripts were quantified by RT-qPCR, and normalized to those of *SCR1*. Depicted are means ± S.D. (N = 3; qPCR = 6). (b) ChIP-qPCR analysis of Pol II within the *YFR057w* promoter and ORF in *sir2Δ* and *SIR^+^* cells exposed to cycloheximide for the indicated times as in A. Pol II occupancy was determined as in Figure 3A. Occupancy of the non-induced promoter (*sir2Δ*) was set to 1.0; all other occupancies (both promoter and ORF) are scaled relative to it. Depicted are means ± S.D. (N = 4; qPCR = 8). (c) ChIP-qPCR analysis of histone PTMs within the *YFR057w* promoter (H3K18ac, H4K16ac, H3K4me2,3) or ORF (H3K79me2) in *sir2Δ* and *SIR^+^* cells exposed for the indicated times to cycloheximide as in A. Shown are normalized PTM/histone H3 quotients. PTM-specific and H3 signals at *YFR057w* were normalized to those measured at *PMA1* and *ARS504*, respectively. The PTM/histone H3 quotient of the non-induced *sir2Δ* sample was set to 1.0 in each case. Depicted are means ± S.D. (N = 2; qPCR = 4). (d) ChIP-qPCR analysis of H3K36me3 enrichment within the ORF and Htz1 enrichment within the promoter as in C, for the indicated times following addition of cycloheximide (N = 2; qPCR = 4). (e) As in D, except H3K56ac enrichment over *YFR057w* promoter and ORF was assayed.

To rule out an Hsf1-specific effect, we asked whether a natural subtelomeric gene could be activated in the absence of covalent histone modification and other chromatin alterations. Previous work has shown that the Sir2/3/4 complex extends ∼3 kb from the right telomere of chromosome VI [61], and that expression of the subtelomeric gene, *YFR057w*, located ∼1 kb from the chromosomal tip, is under *SIR* regulation [62]. RT-qPCR demonstrates that this gene is efficiently silenced by *SIR* (>100-fold; Figure 9A, 0 min). While the function of *YFR057w* is unknown, we reasoned that it might play a role in pleiotropic drug resistance due to the presence of a consensus DNA sequence for Stb5 (www.yeastract.com), which forms a heterodimer with Pdr1. Pdr1 is known to activate other genes involved in pleiotropic drug resistance [e.g., 63]. Consistent with this idea, we found that heterochromatic *YFR057w* was strongly induced by exposure of cells to 200 µg/ml cycloheximide (Figure 9A, black bars). Notably, euchromatic targets of Pdr1 such as *PDR5* and *SNQ2* were also induced by this novel regimen (data not shown), one in which cells remained fully viable (see Figure S2).

The substantial increase in *YFR057w* transcript abundance in the *SIR* ^+^ strain (∼150-fold during first 50 min) is unlikely to be a primary consequence of cycloheximide-mediated mRNA stabilization [64] given that its isogenic *sir2Δ* counterpart showed just a several-fold increase over the same time frame (Figure 9A, white bars). Consistent with bona fide transcriptional induction, Pol II occupancy of the promoter and ORF markedly increased over the cycloheximide time course in both euchromatic and heterochromatic contexts (Figure 9B). The presence of Pol II at the non-induced heterochromatic promoter is consistent with H3 ChIP-Seq data indicating the presence of a 60–80 bp nucleosome-free region upstream of the +1 nucleosome [65].

These striking observations permitted us to ask whether activation of *YFR057w* takes place in the presence of covalent histone modifications, as is the case for euchromatic genes, or in their absence, as is the case for the comparably repressed *hsp82-2001* transgene. We found that induction of heterochromatic *YFR057w* occurred without histone displacement (Figure S3, A and B, black bars) and in the context of minimal nucleosomal alterations including H3K18 and H4K16 acetylation (<5% the level seen at the euchromatic gene (*sir2Δ*; Figures 9C and S3C); H3K4me2,3 and H3K79me2 (<5% of *sir2Δ* context; Figure 9C); H3K36me3 and H2A.Z deposition (20–40% of that seen in a *sir2Δ* context; Figure 9D); and H3K56 acetylation (10–30% of that seen at the euchromatic gene with no detectable enrichment following induction; Figure 9E). Minimal H3K56ac at *YFR057w* is consistent with foregoing results with heterochromatic *hsp82-2001*, which maintained a low level of H3K56ac and a comparatively high nucleosomal density during its activation, although it is unlike observations of a *SIR*-silenced *HMRE*-*GAL10* transgene that suggested acetylation of this lysine is needed for Pol II elongation [43].

Finally, as mentioned above, H4K12 acetylation has been observed at telomeric heterochromatin in *S. cerevisiae*, where it has been implicated in several telomere-related processes including replication, recombination and basal transcription [48]. Indeed, we observed an enrichment of H4K12ac at heterochromatic *YFR057w* relative to other acetylation marks (20–40% of that seen at the euchromatic gene; Figure 5B). Nonetheless, H4K12ac levels remained low in response to cycloheximide induction, a circumstance in which *YFR057w* transcript levels increased several hundred-fold. This, coupled with the fact that heterochromatic *hsp82-2001* contained only background levels of H4K12 acetylation (Figure 5A), argues that this PTM is unlikely to play an important role in heterochromatic gene activation. We conclude that activation of the subtelomeric *YFR057w* gene, like that of *hsp82-2001*, takes place in the context of minimal chromatin modification.

## Discussion

### The Hypoacetylated, Hypomethylated and Nucleosome-Rich Landscape of Activated Silent Chromatin

A widespread belief in the transcription/chromatin field is that covalent modification of nucleosomal histones is integral to the mechanism by which eukaryotes regulate gene expression [5], [9]–[15], [37], [43], [45], [47]–[49], [66]. Results presented here demonstrate that in contrast to this notion, histone modification of activated heterochromatic genes in the model eukaryote *S. cerevisiae* is minimal, and in certain instances undetectable, despite robust induction (>200-fold over the basal silent state) and dynamic occupancy of the transcriptional machinery. Activation with minimal chromatin modification is observed at genes regulated by disparate activators and is seen in distinct genetic backgrounds. It is not the consequence of insubstantial transcription, since the euchromatic gene, *hsp82-ΔTATA*, despite being expressed at a level of only 20–30% of heterochromatic *hsp82-2001*, exhibits striking chromatin modification during its activation including promoter-proximal H3 hyperacetylation and histone displacement. Moreover, at least in the case of *HSP82*, it is not due to a lack of a requirement for covalent histone modifications. Previous work has shown a clear dependence of euchromatic *hsp82* alleles on the histone acetylases Gcn5 and Esa1 present in SAGA and NuA4, respectively [45]. Consistent with this, both *HSP82* and *hsp82-ΔTATA* are extensively acetylated upon heat shock activation and *HSP82* expression is reduced in an H4 N-terminal tail mutant. A similar requirement for histone acetylation likely applies to the euchromatic *YFR057w* gene as its expression is reduced in an H4K16R mutant (Figure S4).

The work presented here significantly extends earlier observations of the absence of tetra-acetylated H4 enrichment following heat shock activation of the *hsp82-2001* transgene [24] in many ways. These include analysis of the coding region of this and other heat shock transgenes, analysis of multiple covalent histone modifications typically linked to gene activation, and most significantly extending the finding to a natural heterochromatic gene, *YFR057w*, whose inducibility was heretofore unknown and whose activation is unlikely to be under the control of Hsf1. It also substantially extends recent observations of a telomere-linked *URA3* gene that like *HSP82* is able to activate in absence of H3 and H4 acetylation [37] (see below).

It is possible that absence of detectable covalent marks is a consequence of their transience. We examined histone PTMs at multiple time points during the transcriptional induction of two unrelated genes, including time points spaced as tightly as 15 sec apart, and could find no compelling evidence for their presence. Nonetheless, our experiments cannot rule out the presence of covalent histone modifications that are erased (or occluded, for example, by “reader” molecules) moments after they appear. However, our inability to detect PTMs is unlikely to be due to masking of stable modifications. We utilized antibodies specific to multiple epitopes within the histone octamer – globular domain of H3, N-terminal epitope of Myc-H4, acidic patch of H2A – and all were readily detectable in SIR-mediated heterochromatin.

In addition to the absence of covalent histone modification, the heterochromatic *hsp82-2001* gene is only partially and transiently depleted of the H3/H4 tetramer during heat shock. This is in contrast to euchromatic *hsp82-2001* that sustains a >90% loss of H2A, H3 and H4 during the early stages (5–25 min) of heat shock induction (Figure 2 and data not shown). Interestingly, even under maximally inducing conditions, the density of H3 and H4 at heterochromatic *hsp82-2001* equals or exceeds its density at the non-induced euchromatic transgene. The continued presence of the Sir complex during heat-induced activation is likely crucial for suppression of both histone loss and covalent histone modifications. Regarding PTMs, in the case of H3/H4 acetylation, the Sir2 deacetylase apparently “wins” the competition with the SAGA and NuA4 acetylases for the chromatin template. Why H3 methylation does not occur is less clear, and is the subject of ongoing investigation.

It is possible that the minimal changes in histone modification state that are detected at silent *hsp82-2001* and *YFR057w* are sufficient to serve as novel surfaces to which bromodomain-, chromodomain- and PHD-domain-containing regulatory complexes may bind. We believe that this is unlikely, even in the extreme case in which it is assumed that the euchromatic counterparts of these genes are saturated with acetylated or methylated histone isoforms at their maximum point of enrichment. Since in most cases there is a >10-fold difference in modification levels in euchromatic (*sir2Δ* or *sir4Δ*) versus heterochromatic (*SIR^+^*) states, modification of the heterochromatic gene is maximally one PTM (of a given type) for every 5–10 nucleosomes. Since the upstream regulatory region of *HSP82* spans only two nucleosomes [67], [68], this means that <1 PTM of a specific type is present within the heterochromatic *hsp82-2001* promoter. This, combined with the fact that there is no evidence for epigenetic variegation of *hsp82-2001* expression (at least when tested under non-inducing conditions [24]), argues against the existence of a subpopulation of cells enriched for activating histone marks and disproportionately contributing to the *HSP82* transcript measured in our assays. Instead, our results suggest a dominant role for gene-specific activators (Hsf1 in the case of *hsp82-2001* and Pdr1/Stb5 in the case of *YFR057w*) in recruiting transcriptional co-regulators [69] that disrupt SIR-mediated silencing.

Our findings also provide an interesting contrast to those of Grunstein, Carey and coworkers [37]. These workers examined the chromatin properties of a telomere-linked *URA3* transgene under control of the Sir2/3/4 chromatin modification complex, both in cell populations in which it was expressed (cells grown in synthetic medium lacking uracil), as well as those in which it was repressed (cells grown in rich medium containing 5-FOA). As was the case here, the expressed state was characterized by substantial levels of Sir3 and deacetylated H3 and H4. In contrast to our findings, however, other histone modifications – in particular, H3K4me3, H3K36me3 and both mono- and di-methylated forms of H3K79 – were abundant [37]. Based on these and other lines of evidence, the authors speculated that the H3K79me2 mark, while not diminishing the abundance of Sir3 at the expressed *URA3-TEL* gene, disrupted its physiological interaction with the underlying nucleosomes, thereby accounting for the transcriptionally permissive template [37]. As our data indicate that both *hsp82-2001* and *YFR057w* can be transcribed essentially in the absence of H3K4, H3K36 and H3K79 methylation, an obligatory role for these histone modifications in promoting Pol II transcription of heterochromatic genes seems unlikely.

Instead, our results are more consistent with a large scale mutational analysis in *S. cerevisiae* which showed that despite the widespread localization of H3K4me, H3K36me and H3K79me marks, deletion of the enzymes responsible for imparting these marks – Set1, Set2 and Dot1 – had very specific effects with the expression of most genes unaffected [70]. Also congruent with our results are observations of *Drosophila* mutants in which essentially normal transcriptional regulation can occur in the complete absence of H3K4 methylation [71].

### A “Fine-Tuning” Role for Histone Modifications in Transcriptional Activation

We have demonstrated that in yeast heterochromatin, substantial increases in transcription can take place in the absence (or near-absence) of chromatin alterations often thought necessary for activation. Nonetheless, gross transcriptional output of *hsp82-2001* and *YFR057w* is reduced several-fold as a consequence of their location within SIR-heterochromatin. Therefore, at least in these two cases, histone modifications may play a role in increasing transcriptional output. Other explanations for how Sir2/3/4 proteins reduce transcription of these genes, including direct or indirect roles in enhancing nucleosome stability [44], are also possible. Whatever the physiological role of histone modifications might be, it is notable that similar to the examples presented here, heat stress-induced activation of heterochromatic transgenes in *Arabidopsis* occurs without reversal of repressive chromatin marks such as DNA methylation and H3K9 and H3K27 methylation, and without the appearance of activating modifications such as H3 and H4 acetylation [72]. Therefore, absence of activating histone modifications may be a common feature of stress-induced heterochromatic transcription.

## Materials and Methods

### Yeast Strains

Strains used in this study are listed in Table 1. The *hsp82* transgenic strains (SLY101 background) were generated by integrating *HMRE* silencers (144 bp modules [separated by 6 bp spacers in the case of tandem integrants]) both 5′ and 3′ of the chromosomal *HSP82* gene using two-step gene transplacement methods [33]. To permit expression of Myc-tagged histone H4, strains were transformed with an episomal *myc-HHF2* gene borne on plasmid pNOY436 (*TRP1-CEN6-ARS4*) as previously described [38]. Strains MSY529 and MSY541 were gifts of M.M. Smith (University of Virginia).

**Table 1:** Yeast strains.

Strain	Genotype	Reference or Source
SLY101	*MATα ade^−^ can1-100 cyh2^r^ his3-11,15 leu2-3,112 trp1-trp1-1 ura3*	[32]
DSG118	SLY101; *hsp82-ΔTATA*	[38]
EAS211	SLY101; *hsp82-201*	[33]
EAS201	EAS211; *sir4Δ::HIS3*	[33]
EAS1011	SLY101; *hsp82-1001*	[33]
EAS1001	EAS1011; *sir4Δ::HIS3*	[33]
EAS2011	SLY101; *hsp82-2001*	[33]
EAS2001	EAS2011; *sir4Δ::HIS3*	[33]
LG110	EAS2011; *htz1Δ::KAN-MX*	This study
LG111	EAS2001; *htz1Δ::KAN-MX*	This study
AJ2001	EAS2001; *dot1Δ::KAN-MX*	This study
AJ2011	EAS2011; *dot1Δ::KAN-MX*	This study
BY4741	*MATa his3*Δ*1 leu2*Δ*0 met15*Δ*0 ura3*Δ0	Research Genetics
LG2883	BY4741; *sir2*Δ::*KAN-MX*	[42]
MSY529	*MATa ura3-52 leu2-3,112 lys2*Δ*201 (hht1 hhf1)*Δ (*hht2 hhf2*)Δ *pMS337(CEN ARS LEU2 HHT1 HHF1)*	[75]
MSY541	MSY529; *pMS385(CEN ARS LEU2 HHT1 hhf1-21)*	[75]
HZY105	MSY529; *sir2Δ::KAN-MX*	This study
HZY106	MSY541; *sir2Δ::KAN-MX*	This study

### Cultivation and Induction Conditions

Yeast strains were cultivated at 30°C to early log phase (A_600_ = 0.3 to 0.7) in either rich yeast extract-peptone-glucose broth supplemented with 0.03 mg/ml adenine or synthetic complete medium lacking tryptophan (SDC-Trp). Heat shock induction was achieved by transferring the culture (typically 50 ml) to a vigorously shaking 39°C water bath; once the temperature reached 39°C, incubation was allowed to continue for an additional 20 min before addition of either sodium azide (to a 20 mM final concentration (RNA assays)) or formaldehyde (to a 1% final concentration (ChIP)). For time course assays, instantaneous 30° to 39°C upshift was achieved by rapidly mixing equal volumes of 30°C culture and pre-warmed medium (55°C) and then incubating with rapid shaking at 39°C for the times indicated. Induction was terminated through addition of sodium azide.

For drug induction, 100 ml early log cultures (A_600_ ∼0.6) were made 200 µg/ml in cycloheximide through a 1∶100 dilution of a 20 mg/ml stock solution prepared in DMSO. Cells were then incubated with agitation at 30°C for various times (indicated in Figure legends) prior to addition of 20 mM sodium azide. Cells were then harvested, washed in 20 mM azide, and stored at −80°C for subsequent chromatin or RNA isolation.

### ChIP Analysis

End point (gel-based) ChIP-PCR analysis (Figures 2A, 3B, 4B, 4C, 4E–G, 7A, S1A) was conducted essentially as described [42]. Briefly, 50 ml of mid-log culture (A_600_ ∼0.5) were cross-linked with 1% formaldehyde for 10 min, then converted to spheroplasts with lyticase (4 mg/ml; Sigma). Spheroplasts were lysed using one volume of 0.5 mm glass beads for 30 min at 4°C on an Eppendorf 5432 mixer. Chromatin was sheared to a mean size of ∼0.5 kb with a Branson 250 sonifier equipped with a microtip using three 25 s pulses at constant power and an output setting of 22 watts. The clarified supernatant (final volume 3.0 ml) was used in immunoprecipitations (IPs) that were typically achieved by adding 2–5 µl antiserum to 300 µl of chromatin lysate. Signal quantification was done using a Storm 860 PhosphorImager (Molecular Dynamics) and ImageQuant 5.2 software. To calculate the relative abundance of a given gene sequence present in an IP, we used the following formula: Q_gene_ = IP_gene_/Input_gene_. In most cases, the abundance of each test locus was expressed relative to that of either *PHO5* or *ARS504*, which served as internal recovery controls. In the case of Sir3, abundance at a given locus was quantified relative to its abundance at *HMRa1*. To reduce background, we subtracted the signal arising from a mock IP (-Ab) for the histone covalent modification, Htz1 and Cet1 ChIPs, and the signal arising from pre-immune serum for the Pol II (Rpb1) ChIPs. For the nonspecific *ARS504* IP signal, only gel background was subtracted.

Real Time (ChIP-qPCR) analysis (Figures 2B, 2C, 3A, 5, 6, 8[B–F], 9[B–E], S3) was conducted as follows. Briefly, 125 ml of mid-log cell culture were used and 25 ml aliquots were removed for each time point. Two ml crosslinked chromatin were obtained from each, and ∼10% of that (200–250 µl) was employed for each IP. For all ChIP-qPCR assays, chromatin was isolated as above except cells were lysed with glass beads in the presence of 1% Triton X-100 and 0.1% sodium deoxycholate. Immunoprecipitations were conducted through addition of 40 µl of a 50% slurry of CL-4B Protein A Sepharose beads, with incubation at 4°C overnight. Following immunoprecipitation, DNA was purified and dissolved in 60 µl TE; 2 µl of immunoprecipitated DNA added to each 20 µl Real Time PCR reaction. This was performed on an Applied Biosystems 7900HT Real-Time PCR system using RT^2^ qPCR SYBR Green/ROX MasterMix (SABiosciences; #330529). Through use of a standard curve specific for each amplicon, the quantity of DNA present in each IP was determined, and background signal was subtracted. In the case of Myc ChIPs, the background was the signal arising from chromatin isolated in a parallel culture of an isogenic strain lacking Myc-tagged H4; for histone PTM ChIPs, background was the signal arising from a beads alone control. For Pol II ChIP, background was the signal arising from pre-immune serum. To normalize for variation in sample recovery, abundance of a non-transcribed region on chromosome V (*ARS504*) was determined for each ChIP DNA sample, and *HSP82/ARS504* and *YFR057w/ARS504* quotients were derived. In certain cases (see figure legends), normalization to the *PHO5* promoter was done instead. Finally, to account for nucleosome loss, all PTM data are presented as (histone PTM)/Myc-H4 or (histone PTM)/H3 quotients.

The following antibodies were used: Myc (MAb 9E10, Santa Cruz Biotechnology); H3 globular domain (ab1791, Abcam); H3 K9ac, K14ac (12-360; Millipore); H3 K18ac (ab1191; Abcam); H3 K56ac (gift of M. Grunstein, UCLA); H4 K16ac (07-329, Millipore); H4 K12ac (07-595, Millipore); H4 tetra-acetylated (K5, K8, K12, K16) (06-866, Millipore); H2A acidic patch (residues 88-97; 07-146, Millipore); H3 K4me3 (ab8580; Abcam); H3 K4me2,3 (ab6000; Abcam); H3 K36me3 (ab9050; Abcam); H3 K79me2 (ab3594; Abcam); Htz1 (residues 1–100; ab4626; Abcam); Cet1 (gift of S. Buratowski, Harvard Medical School); Sir3 (gift of R.T. Kamakaka, University of California, Santa Cruz); Pol II, rabbit antiserum raised in our laboratory against a recombinant GST-CTD polypeptide bearing 52 heptad repeats of the mouse large subunit [38].

Amplicons for endpoint PCR were as follows (coordinates relative to ATG): *HSP82* promoter, −401 to −34; *HSP82* ORF, +1248 to +1444; *HSP82* 3′-UTR, +1883 to +2155; *ARS504*, coordinates 9746 to 9817; *PHO5* promoter, −507 to −33; *CIN2* 5′ ORF, +149 to +427; *CIN2* 3′-UTR, +523 to +797; *YAR1* ORF, +3 to +200; *YAR1* 3′-UTR, +1109 to +1297); *IQG1* 5′ ORF, +1016 to +1211; *IQG1* 3′ ORF, +2007 to +2289; *SUI3*, +451 to +748; *YPL236c*, +71 to +229; *HMRa1*, −80 to +50. For Real Time PCR, the amplicons were: *HSP82* UAS, −227 to −140; *HSP82* promoter, −157 to −88 or −227 to −88 (Figures 2B, 2C, 6C–E); *HSP82* ORF, +1248 to +1444; *HSP82* 3′-UTR, +2134 to +2228; *YFR057w* promoter, −115 to −45; *YFR057w* ORF, +312 to +437; *ARS504*, coordinates 9746 to 9817; *PHO5* promoter, −197 to −124; *PMA1* 5′-coding region, +49 to +112.

### Northern Analysis

For the expression analyses summarized in Figure 1B and illustrated in Figures 4D and S1[B-E], RNA was isolated from 10 ml aliquots of cell culture employed for ChIP assays using the glass bead lysis method [73], and blots were hybridized to a gene-specific probe, exposed to PhosphorImager, then re-hybridized to an *ACT1* probe as done previously [42]. Probes used were as follows: *HSP82*, +2167 to +2228; *CIN2*, +149 to +427; *YAR1*, +3 to +200; *SUI3*, +451 to +748; *IQG1*, +1016 to +1211; and *ACT1*, +606 to +1000.

### RT-qPCR Analysis

For the expression analyses illustrated in Figures 4A, 7B and 8A, cells were cultivated to an A_600_ of ∼0.5 in a 125 ml culture at 30°C and 15 ml aliquots were removed and subjected to an instantaneous 30 to 39°C upshift for the indicated times. Heat shock induction was terminated through addition of 20 mM sodium azide, and RNA was isolated as above. For induction of *YFR057w* (Figures 9A, S4), cycloheximide was added to a final concentration of 0.2 mg/ml, 15 ml aliquots were removed at the indicated times and RNA was isolated.

Contaminating genomic DNA was removed from each RNA sample by digestion with RNase-free DNase I (OMEGA Bio-tek Inc #E1091), followed by phenol/chloroform extraction. 0.5 µg purified RNA was used in each cDNA synthesis with ProtoScript RT-PCR Kit (NEB #E6400S). Oligo(dT) primers were used in cDNA synthesis for quantification of Pol II gene transcripts; random primers were used in cDNA synthesis for quantification of *SCR1* RNA. 2% of the synthesized cDNA was used in each qPCR, which was performed as described above. Primers were designed to target the 3′UTR of *HSP82, YFR057w* and *PMA1*, or the body of *SCR1*. Their coordinates are: *HSP82*, +2134 to +2228; *YFR057w*, +312 to +437; *PMA1*, +2998 to +3083 and *SCR1*, +385 to +483. For quantification, *SCR1* was used to normalize *HSP82* and *YFR057w* mRNA levels.

## Supporting Information

Figure S1 The domain of SIR silent chromatin spans at least 4 kb at the silencer-bracketed transgenes *hsp82-1001* and *hsp82-2001*, while it is restricted to ∼1.5 kb at *hsp82-201* that bears tandem silencers upstream of the gene. (a) Sir3 occupancy within each transgenic locus relative to its occupancy at *HMRa1*. ChIP analysis of *SIR^+^* strains bearing the indicated *hsp82* allele was conducted and quantified as described in Figure 2A. Cells were cultivated at 30°C and either maintained at that temperature (−) or heat shocked at 39°C for 20 min (+). Midpoint coordinates are indicated for each amplicon. *, location of integrated silencers (see Figure 1A). Depicted is a summary of three independent experiments (means ± S.E.). (b–e) Northern analysis of *YAR1*, *CIN2*, *SUI3* and *IQG1* in the parent *HSP82* ^+^ strain (SLY101) and the indicated *SIR^+^* and *sir4Δ* transgenic strains. Mean transcript abundance of each gene (normalized to *ACT1*) is quantified relative to that present in the parent strain, which was arbitrarily set to100 (N = 2).
(PDF)

Figure S2 Cycloheximide viability assay. Isogenic *SIR^+^* and *sir2Δ* cells (strains BY4741 and LG2883, respectively) were grown to mid-log (A_600_ = 0.3) in rich YPDA medium, then cycloheximide was added to a final concentration of 200 µg/ml and cells were cultivated at 30°C for the indicated times. Aliquots were removed, diluted 1∶400 in sterile water, and 10 µl were spread onto YPDA plates. Cells were incubated at 30°C for 2.5 days.
(PDF)

Figure S3 Activation of the heterochromatic *YFR057w* gene occurs without detectable nucleosomal disruption or H3/H4 acetylation. (a) H3 occupancy of the *YFR057W* promoter in *sir2Δ* and *SIR^+^* cells at the indicated times following addition of 200 µg/ml CX to mid-log cultures. H3 levels were normalized to those at *PHO5*. Depicted are means ± S.D. (N = 2; qPCR = 4). (b) H2A occupancy of the *YFR057W* promoter conducted and quantified as in A. (c) H3K18ac, H4K16ac and H4 tetra-acetylated ChIP analysis of the *YFR057w* promoter and ORF in *sir2Δ* or *SIR^+^* cells exposed to CX for the indicated times. H3 and each PTM were normalized to their occupancy at *ARS504*, and then the PTM/H3 quotient of the non-induced (0 min) *sir2Δ* sample was set to 1.0. Depicted are means ± S.D. (N = 2; qPCR = 4).
(PDF)

Figure S4 Transcriptional activation of the euchromatic *YFR057w* gene is impeded in an H4 K16R mutant. Depicted is *YFR057w* transcript abundance in *sir2Δ* derivatives of MSY529 and MSY541. Cells were incubated for the indicated times in the presence of cycloheximide (CX), RNA isolated and *YFR057w* RNA levels determined as in Figure 9A. Depicted are means ± S.D. (N = 2; qPCR = 4).
(TIF)
