# Supplementary figures and images for: Uncoupling Transcription from Covalent Histone Modification
Source: PLoS Genet. 2014 Apr 10;10(4):e1004202. doi: 10.1371/journal.pgen.1004202 (PMC3983032; doi:10.1371/journal.pgen.1004202)

**A**

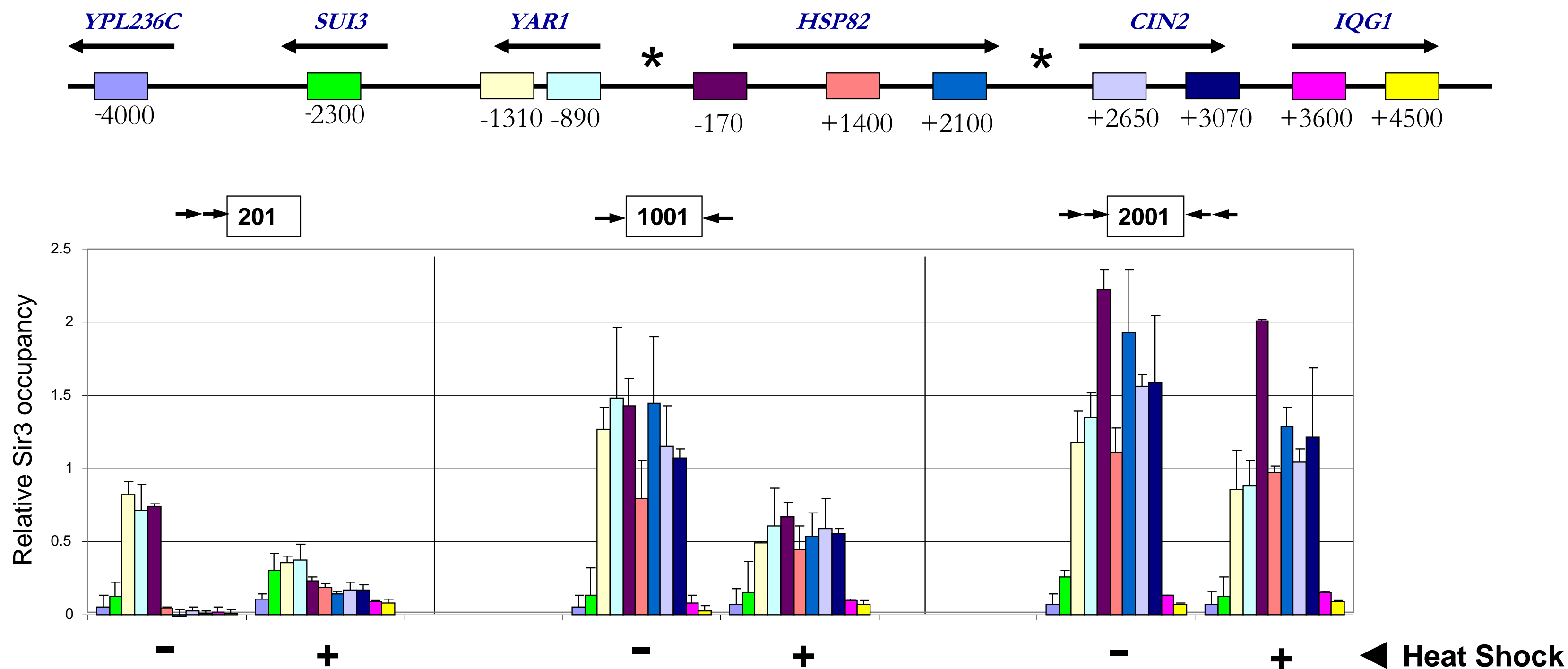

**B**

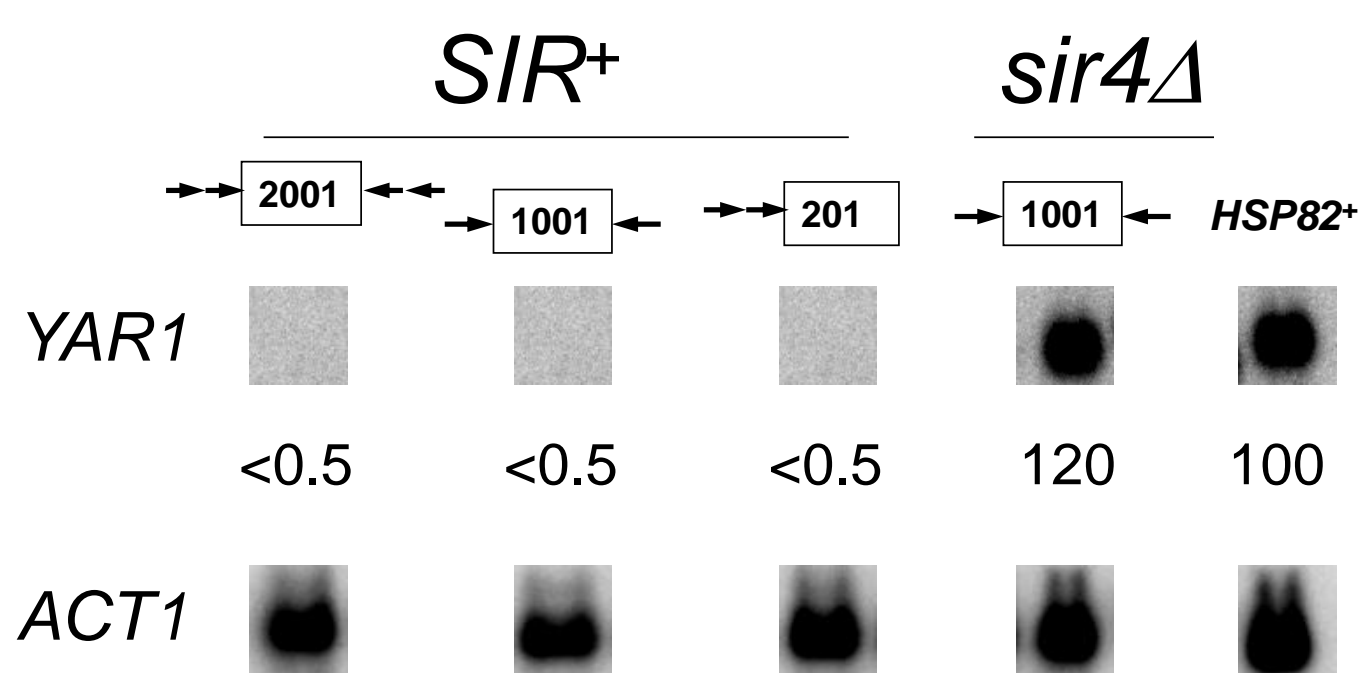

**C**

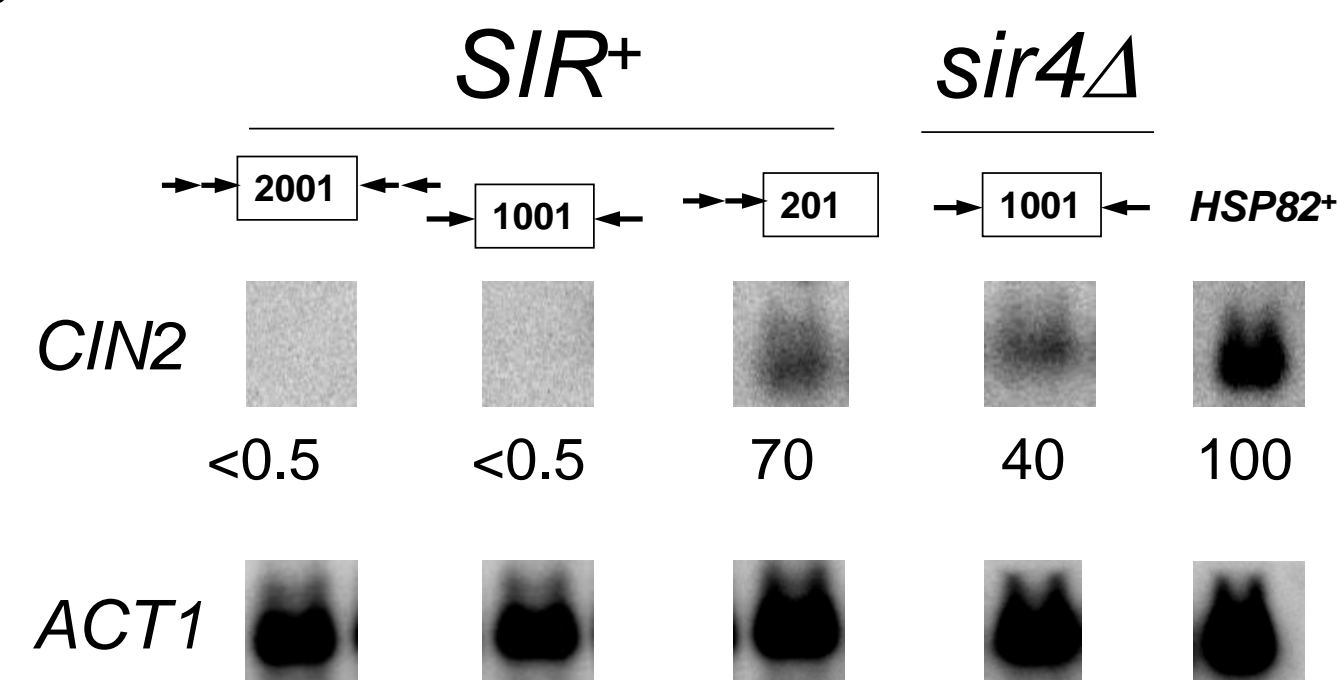

**D**

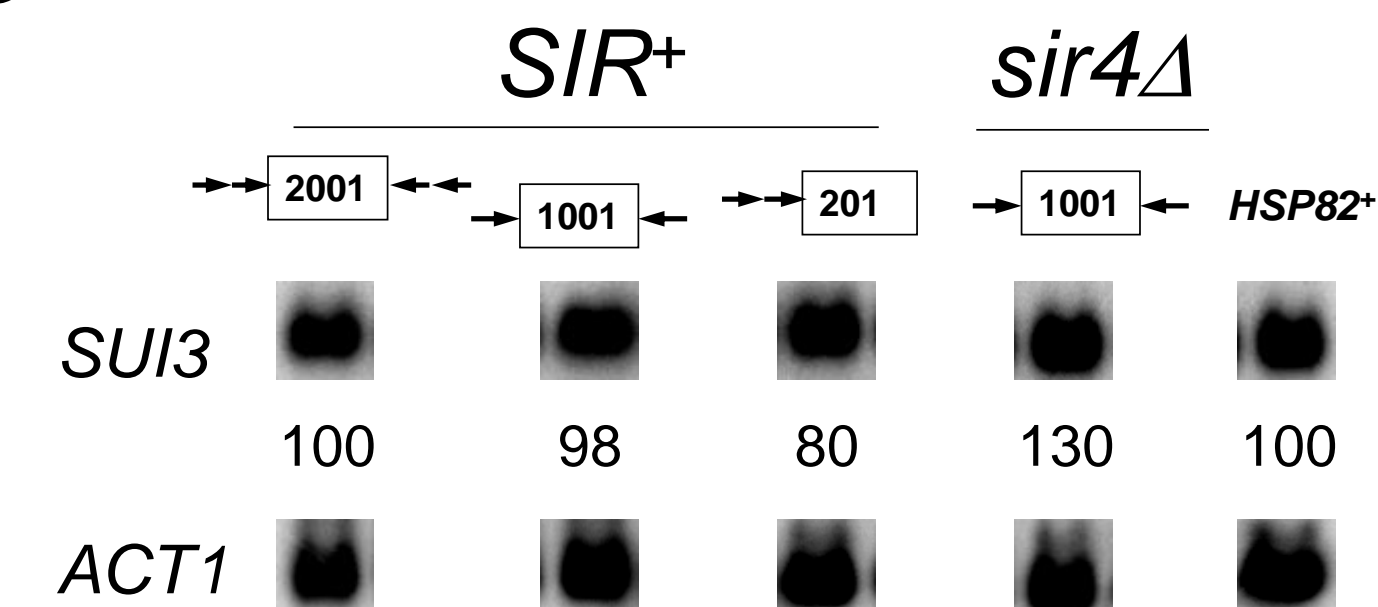

**E**

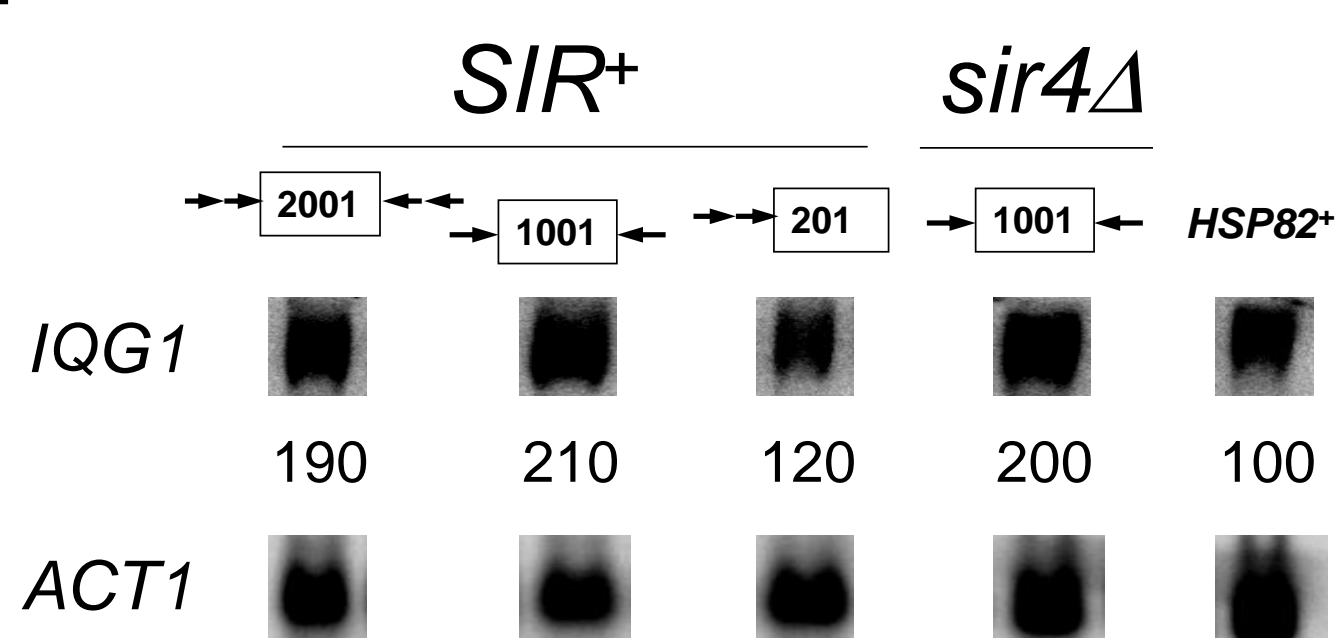

Supplement: Figure S1 — The domain of SIR silent chromatin spans at least 4 kb at the silencer-bracketed transgenes hsp82-1001 and hsp82-2001, while it is restricted to ∼1.5 kb at hsp82-201 that bears tandem silencers upstream of the gene. (a) Sir3 occupancy within each transgenic locus relative to its occupancy at HMRa1. ChIP analysis of SIR+ strains bearing the indicated hsp82 allele was conducted and quantified as described in Figure 2A. Cells were cultivated at 30°C and either maintained at that temperature (−) or heat shocked at 39°C for 20 min (+). Midpoint coordinates are indicated for each amplicon. *, location of integrated silencers (see Figure 1A). Depicted is a summary of three independent experiments (means ± S.E.). (b–e) Northern analysis of YAR1, CIN2, SUI3 and IQG1 in the parent HSP82 + strain (SLY101) and the indicated SIR+ and sir4Δ transgenic strains. Mean transcript abundance of each gene (normalized to ACT1) is quantified relative to that present in the parent strain, which was arbitrarily set to100 (N = 2). (PDF) [file pgen.1004202.s001.pdf]

*SIR*<sup>+</sup>

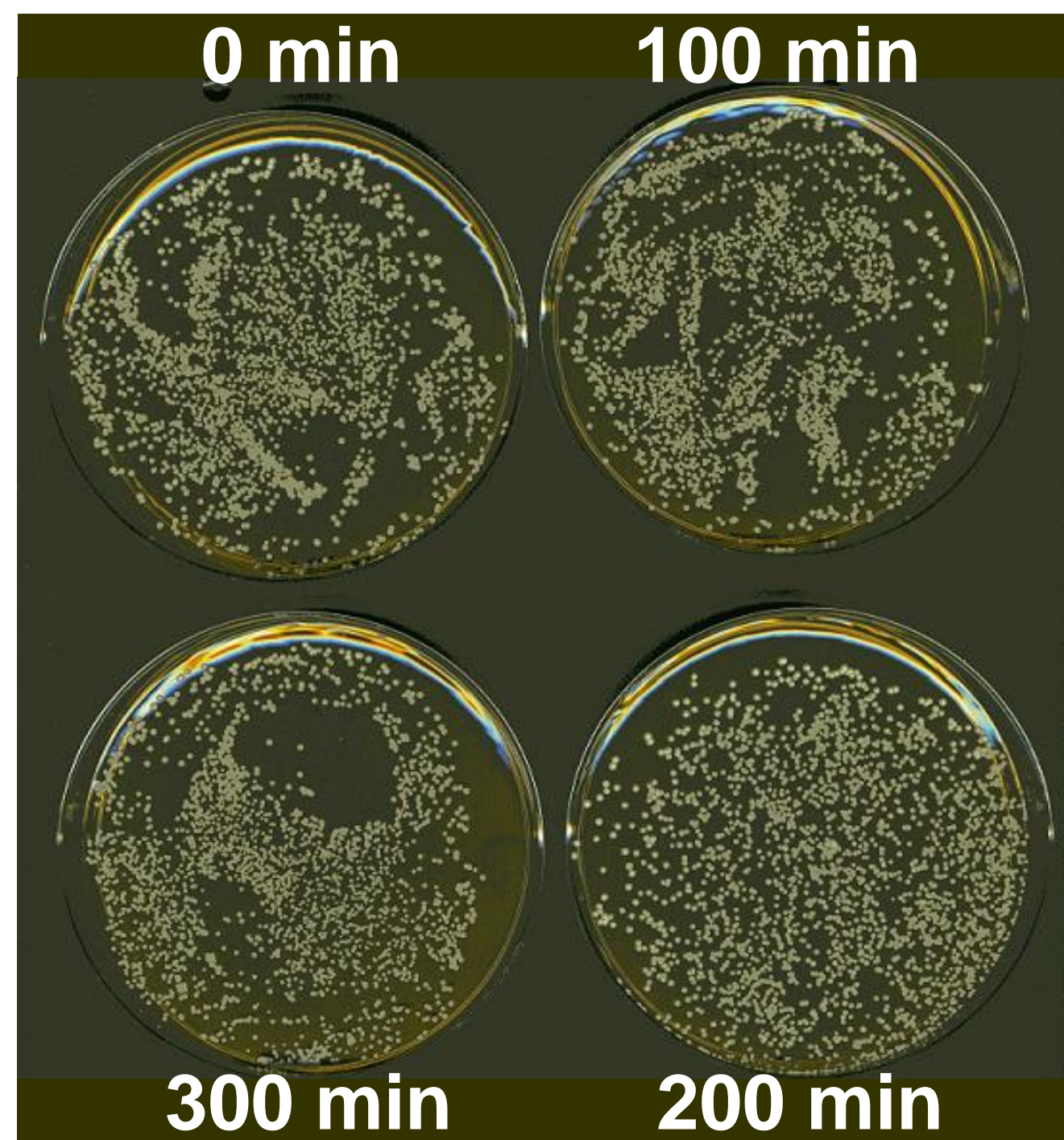

*sir2*Δ

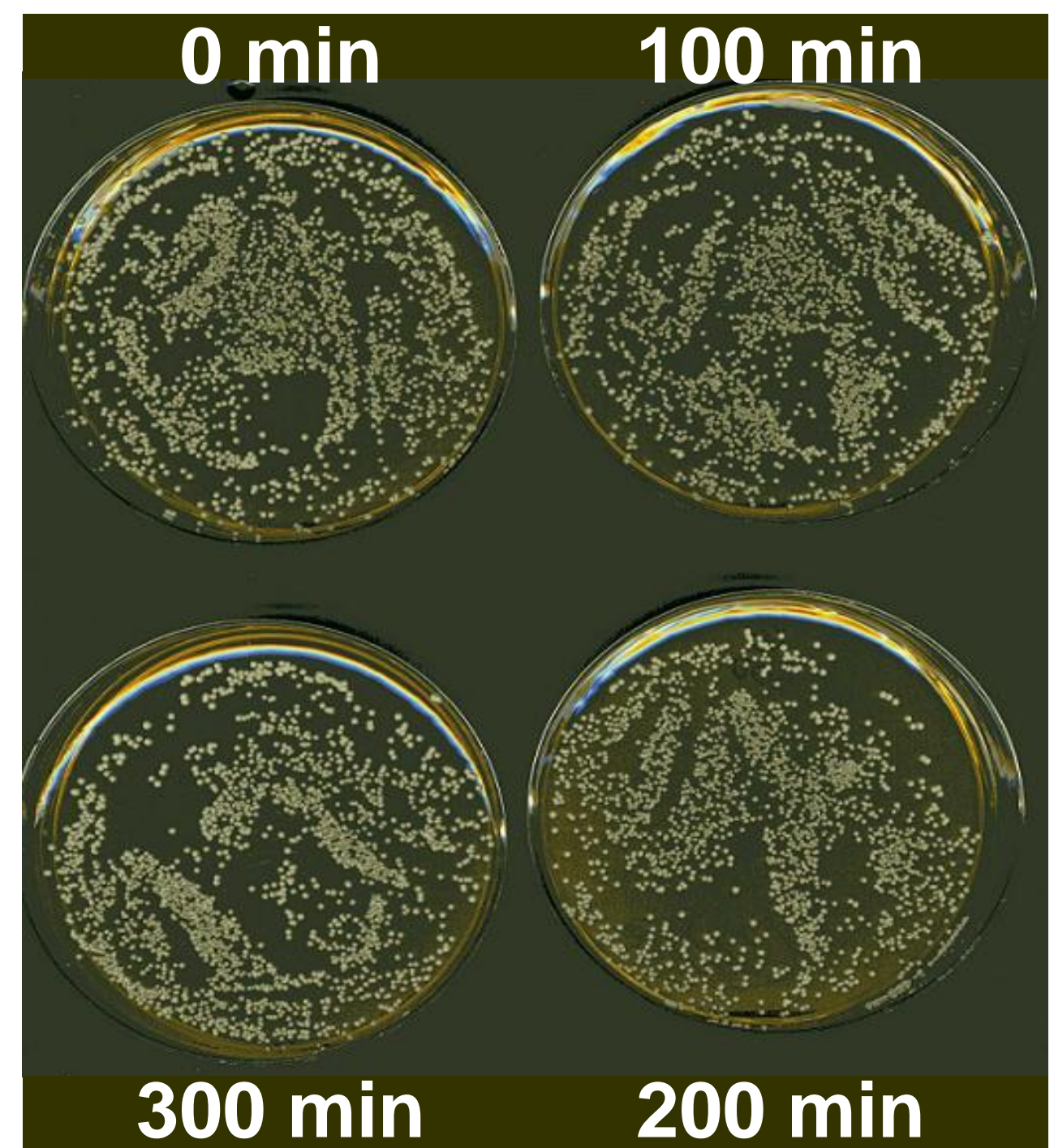

Supplement: Figure S2 — Cycloheximide viability assay. Isogenic SIR+ and sir2Δ cells (strains BY4741 and LG2883, respectively) were grown to mid-log (A600 = 0.3) in rich YPDA medium, then cycloheximide was added to a final concentration of 200 µg/ml and cells were cultivated at 30°C for the indicated times. Aliquots were removed, diluted 1∶400 in sterile water, and 10 µl were spread onto YPDA plates. Cells were incubated at 30°C for 2.5 days. (PDF) [file pgen.1004202.s002.pdf]

# YFR057w

**A**

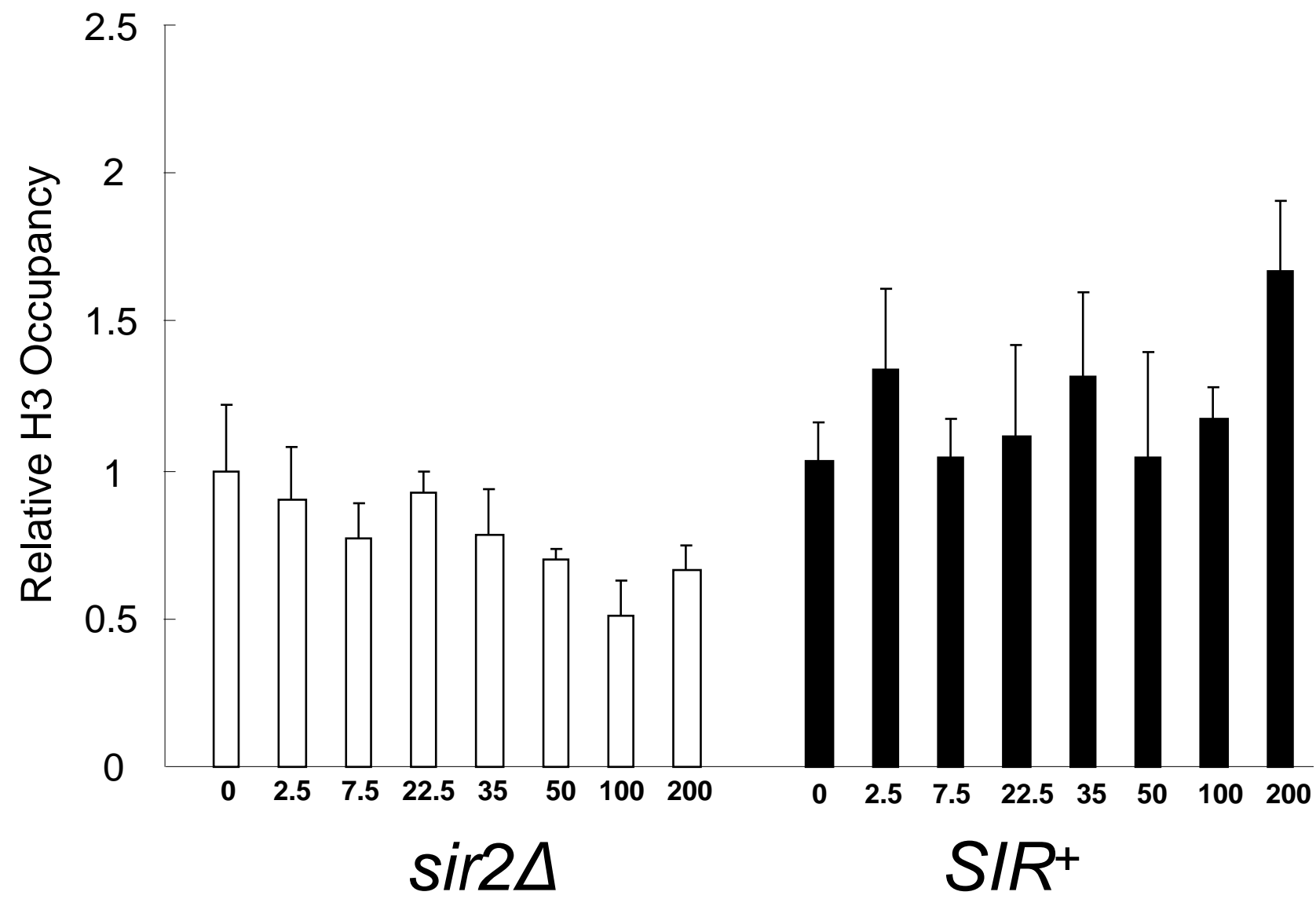

**B**

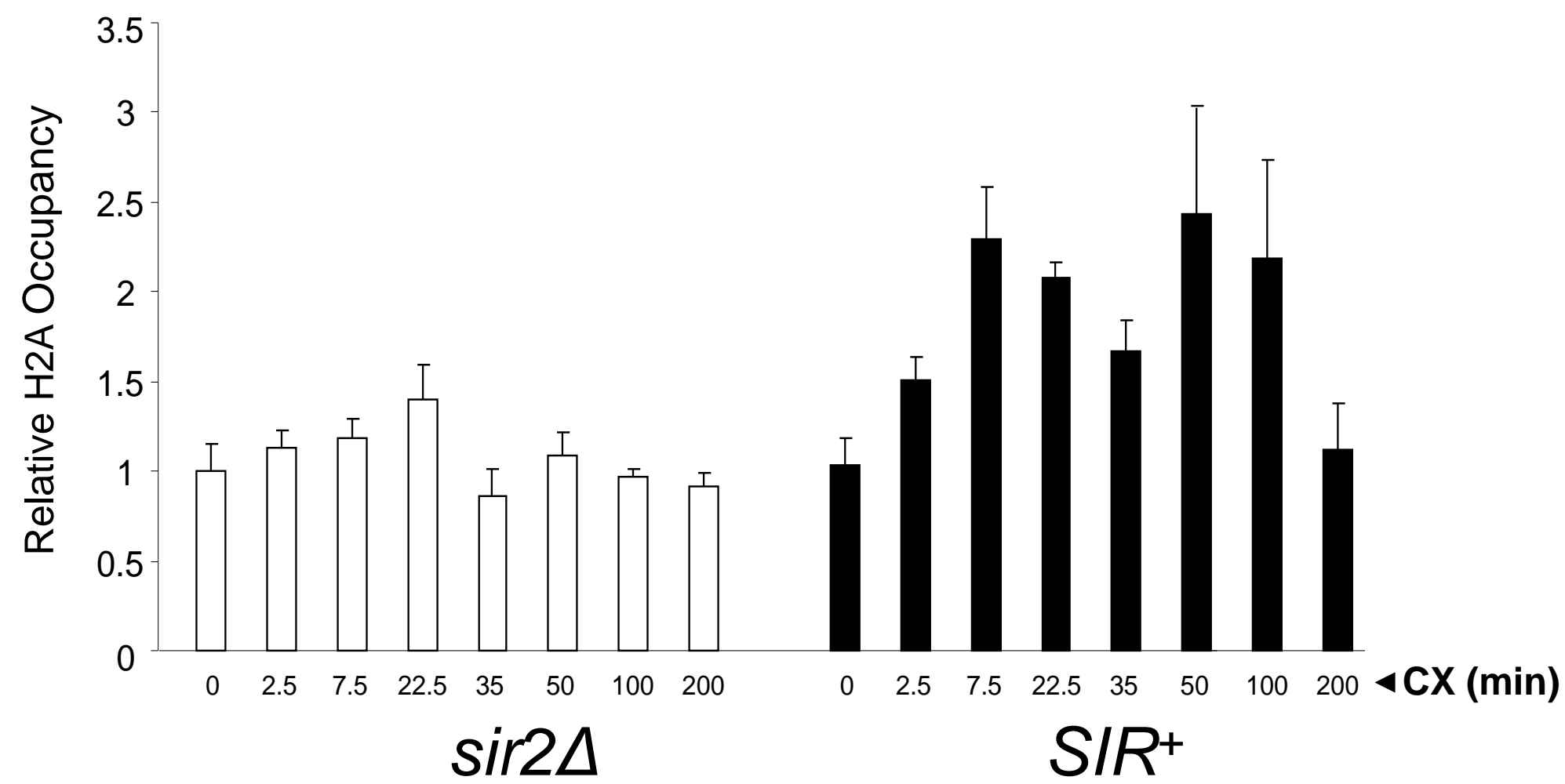

**C**

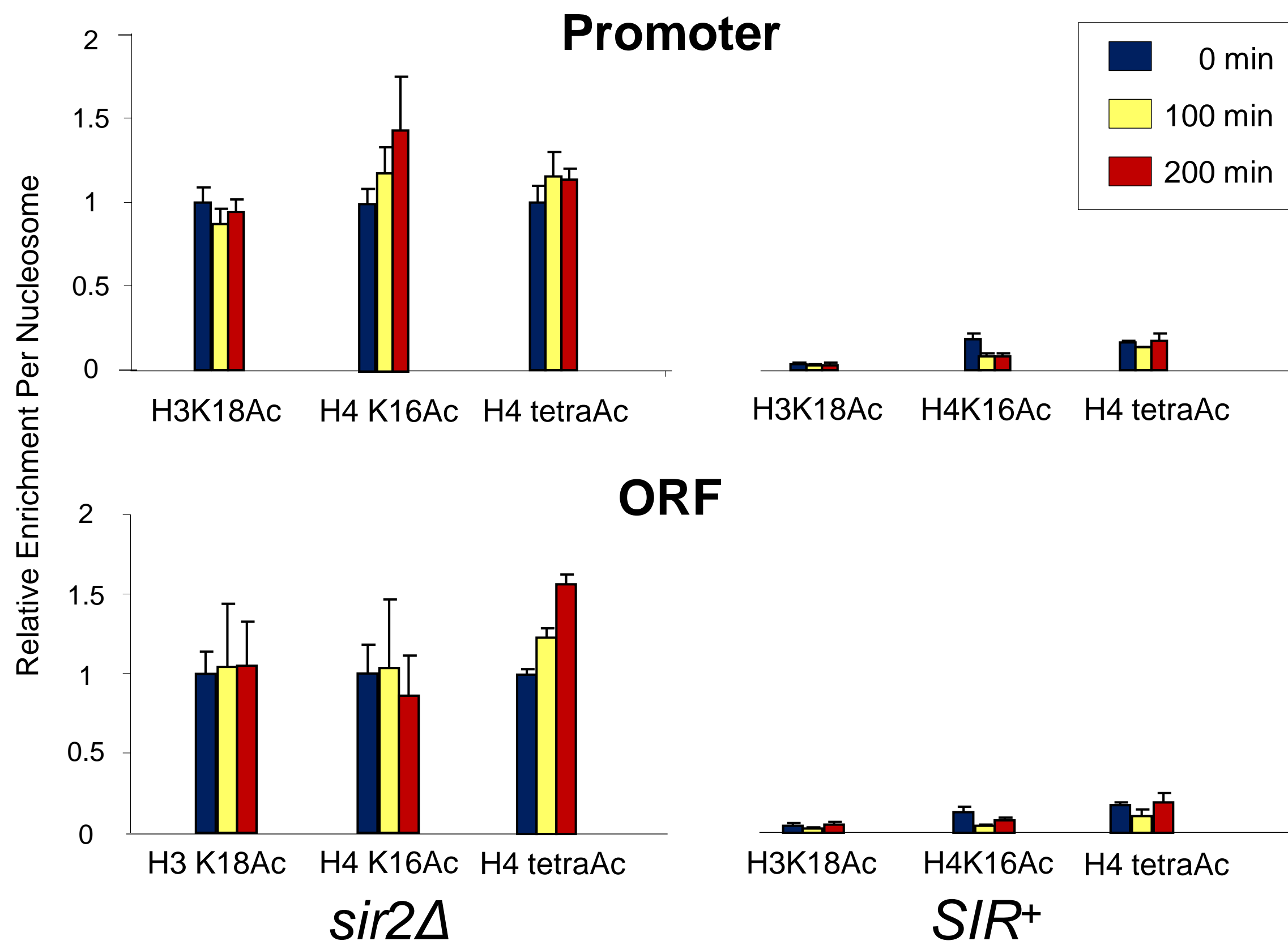

Supplement: Figure S3 — Activation of the heterochromatic YFR057w gene occurs without detectable nucleosomal disruption or H3/H4 acetylation. (a) H3 occupancy of the YFR057W promoter in sir2Δ and SIR+ cells at the indicated times following addition of 200 µg/ml CX to mid-log cultures. H3 levels were normalized to those at PHO5. Depicted are means ± S.D. (N = 2; qPCR = 4). (b) H2A occupancy of the YFR057W promoter conducted and quantified as in A. (c) H3K18ac, H4K16ac and H4 tetra-acetylated ChIP analysis of the YFR057w promoter and ORF in sir2Δ or SIR+ cells exposed to CX for the indicated times. H3 and each PTM were normalized to their occupancy at ARS504, and then the PTM/H3 quotient of the non-induced (0 min) sir2Δ sample was set to 1.0. Depicted are means ± S.D. (N = 2; qPCR = 4). (PDF) [file pgen.1004202.s003.pdf]
